# Phytocannabinoid‐dependent mTORC1 regulation is dependent upon inositol polyphosphate multikinase activity

**DOI:** 10.1111/bph.15351

**Published:** 2021-01-18

**Authors:** Joseph L. Damstra‐Oddy, Eleanor C. Warren, Christopher J. Perry, Yann Desfougères, John‐Mark K. Fitzpatrick, Judith Schaf, Lisa Costelloe, William Hind, Eric J. Downer, Adolfo Saiardi, Robin S.B. Williams

**Affiliations:** ^1^ Centre for Biomedical Sciences, School of Biological Sciences Royal Holloway University of London Egham UK; ^2^ Laboratory for Molecular Cell Biology University College London London UK; ^3^ Discipline of Physiology, School of Medicine, Trinity Biomedical Sciences Institute, Trinity College Dublin University of Dublin Dublin Ireland; ^4^ Department of Neurology Beaumont Hospital Dublin Ireland; ^5^ GW Research Ltd Histon UK

**Keywords:** cannabidiol, cannabigerol, IPMK, mTORC1, multiple sclerosis

## Abstract

**Background and Purpose:**

Cannabidiol (CBD) has been shown to differentially regulate the mechanistic target of rapamycin complex 1 (mTORC1) in preclinical models of disease, where it reduces activity in models of epilepsies and cancer and increases it in models of multiple sclerosis (MS) and psychosis. Here, we investigate the effects of phytocannabinoids on mTORC1 and define a molecular mechanism.

**Experimental Approach:**

A novel mechanism for phytocannabinoids was identified using the tractable model system, *Dictyostelium discoideum*. Using mouse embryonic fibroblasts, we further validate this new mechanism of action. We demonstrate clinical relevance using cells derived from healthy individuals and from people with MS (pwMS).

**Key Results:**

Both CBD and the more abundant cannabigerol (CBG) enhance mTORC1 activity in *D. discoideum*. We identify a mechanism for this effect involving inositol polyphosphate multikinase (IPMK), where elevated IPMK expression reverses the response to phytocannabinoids, decreasing mTORC1 activity upon treatment, providing new insight on phytocannabinoids' actions. We further validated this mechanism using mouse embryonic fibroblasts. Clinical relevance of this effect was shown in primary human peripheral blood mononuclear cells, where CBD and CBG treatment increased mTORC1 activity in cells derived from healthy individuals and decreased mTORC1 activity in cells derived from pwMS.

**Conclusion and Implications:**

Our findings suggest that both CBD and the abundant CBG differentially regulate mTORC1 signalling through a mechanism dependent on the activity of the upstream IPMK signalling pathway, with potential relevance to the treatment of mTOR‐related disorders, including MS.

**Abbreviations**
4EBP1: eukaryotic translation initiation factor 4E‐binding protein 1
IP_6_
: inositol hexaphosphate
IPMK: inositol polyphosphate multikinase
MS: multiple schlerosis
mTOR (C1/2): mechanistic target of rapamycin (complex 1/2)
PBMCs: peripheral blood mononuclear cells
pwMS: people with MS
REMI: restriction enzyme‐mediated insertion
RIPA: radioimmunoprecipitation assay buffer

**What is already known**

- Deregulated mTORC1 signalling is found in numerous disorders (mTORopathies) including multiple sclerosis, epilepsy and cancer
- Specific phytocannabinoids show both inhibitory and activatory effects on mTORC1 without a known mechanism

**What this study adds**

- We show that specific phytocannabinoids (cannabidiol, cannabigerol) differentially regulate mTORC1 dependent upon IPMK function
- We demonstrate these mechanisms in primary PBMCs derived from pwMS

**What is the clinical significance**

- Investigating IPMK signalling in mTORopathy‐related diseases may provide new insight to disease function
- Cannabigerol, a largely unexplored phytocannabinoid, may provide a new therapeutic approach for mTORopathy treatment

## 1 INTRODUCTION

Spasticity associated with multiple sclerosis (MS) (Zettl, Rommer, Hipp, & Patejdl, 2016) can be treated with a combination of phytocannabinoids, containing the non‐euphoric compound cannabidiol (CBD) and the euphoric Δ^9^‐tetrahydrocannabinol (THC). CBD and THC represent the most studied of over 100 phytocannabinoids and a range of molecular studies has suggested various mechanisms of action for them (Friedman, French, & Maccarrone, 2019). In contrast, cannabigerol (CBG) is one of the most abundant phytocannabinoids found in the cannabis plant (Swift, Wong, Li, Arnold, & McGregor, 2013) and represents a relatively unstudied compound without indication of efficacy in MS treatment or insight to molecular mechanisms.

Conflicting studies have proposed that CBD either enhances or reduces mechanistic target of rapamycin complex 1 (mTORC1) signalling in preclinical models. For example, a mouse model of MS shows reduced mTOR activity in neurons and CBD treatment restored mTOR signalling (Giacoppo, Pollastro, Grassi, Bramanti, & Mazzon, 2017), yet mTOR is activated in immune cells in this disease and reducing mTORC1 signalling provides a therapeutic mechanism (Mammana et al., 2018). Similarly, in a rat model of induced schizophrenia, mTOR signalling is reduced (Renard et al., 2016), whereas CBD treatment increased mTOR activity and reversed symptoms. In contrast, several preclinical studies focused on tuberous sclerosis complex (TSC)‐associated epilepsy and breast cancer, showing pathology‐associated elevated mTORC1 activity and showing that CBD treatment reduced pathway activity (Serra et al., 2019; Sultan, Marie, & Sheweita, 2018). Despite numerous suggested mechanisms for CBD on the G protein‐coupled receptor 55 (GPR55) (Kaplan, Stella, Catterall, & Westenbroek, 2017), adenosine signalling (Liou et al., 2008), transient receptor potential vanilloid type 1 (TRPV1) (Costa, Giagnoni, Franke, Trovato, & Colleoni, 2004) and the folate one‐carbon cycle (Perry, Finch, Muller‐Taubenberger, Leung, & Williams, 2019), these mechanisms do not provide direct insight to the effect of these phytocannabinoids on mTOR signalling.

The model system *Dictyostelium discoideum* has been instrumental in identifying molecular mechanisms of action of a range of drugs and natural products (Chang et al., 2012; Kelly, Sharma, Wilkinson, & Williams, 2018; Perry et al., 2019). This organism contains many human protein orthologues and conserved signalling pathways linked to disease‐related processes (Schaf, Damstra‐Oddy, & Williams, 2019) and enables a range of advantageous research approaches, including the ability to screen mutant libraries providing an unbiased approach to identifying cellular mechanism of action of drugs (Chang et al., 2012; Kelly et al., 2018; Schaf et al., 2019), including effects of CBD on cell growth and folate one‐carbon metabolism (Perry et al., 2019). Characterisation of related molecular effects and restoration of sensitivity by the expression of human orthologous proteins (Chang et al., 2012; Kelly et al., 2018; Perry et al., 2019; Schaf et al., 2019) provide insight to the mechanism of action of the compound translatable to mammalian models (Chang et al., 2012; Chang et al., 2013; Chang et al., 2015; Kelly et al., 2018; Perry et al., 2019; Schaf et al., 2019).

In this study, we investigate the mechanism and specificity of phytocannabinoids in mTORC1 regulation. We show that in *D. discoideum*, both CBD and CBG enhance mTORC1 activity and, focusing on CBG, we identify that genetic changes leading to increased expression of inositol polyphosphate multikinase (IPMK) reduce the CBG‐dependent inhibitory effect on growth. Surprisingly, we show that increased expression of *D. discoideum* or human IPMK elevates mTORC1 activity but inverts the effect of CBG and CBD on mTORC1, resulting in a CBG‐ and CBD‐dependent reduction in mTORC1 activity. We then confirm a role for IPMK in regulating mTORC1 activity in both mouse embryonic fibroblasts and in a clinical setting, where peripheral blood mononuclear cells (PBMCs) derived from healthy individuals show an increased mTORC1 activity upon treatment with CBG, CBD and a mixture of CBD and THC. Conversely, cells from people with MS (pwMS) show elevated mTORC1 activity that is reduced following phytocannabinoid treatment.

## 2 METHODS

### 2.1 Western blot analysis of mTOR activity: WT (AX3), *IPMK* ^*+*^, *PKBA* ^*−*^ and *PKB* ^*−*^ */PKGB* ^*−*^

Briefly, *D. discoideum* cells were treated with purified plant‐derived CBG, CBD, cannabidiolic acid (CBDA) and cannabidivarin (CBDV) (phytocannabinoids at concentration of 0.25 μM; GW Research Ltd, Cambridge, UK) or a PI3K inhibitor (60 μM) (LY294002, Cambridge Bioscience, CAY70920) for 1 h or with a vehicle (−) dimethyl sulfoxide (DMSO)‐only control (0.2%). Protein extracts were separated by SDS‐PAGE gel electrophoresis, transferred onto a PVDF membrane (Fisher Scientific, 88520), analysed for p4EBP1 levels (Cell Signalling Technology, 9459) and imaged using a horseradish peroxidase conjugated secondary antibody in combination with an Odyssey Chemifluorescent Substrate Kit (LI‐COR, 928‐30005). This antibody showed specific binding to both *D. discoideum* and human proteins of the correct molecular weight (Figure S1). MCCC1 was used as a loading control (1:5000 Streptavidin, Alexa Fluor™ 680 conjugate, Thermo Fisher, S21378) as previously reported (Davidson, King, & Insall, 2013). For GefS levels, PVDF membranes (Millipore, IPFL00010) were probed for p‐PKB substrate (Cell Signaling Technology Cat# 9614, RRID:AB_331810) and imaged using a IRDye® 800CW goat anti‐rabbit IgG secondary antibody (LI‐COR, 926‐32211, RRID:AB_621843). In these and the following *D. discoiduem* and MEF experiments, samples were randomly assigned to different treatments and all test groups were included in each experiment.

### 2.2 Growth and development assay of *D. discoideum*: WT (AX3), *IPMK* ^*+*^, human *IPMK* ^*+*^, *PKBA* ^*−*^ and *PKBA* ^*−*^ *IPMK* ^*+*^

In summary, *D. discoideum* cells were grown in axenic medium (Formedium, HLB0103) containing 100 μg/ml penicillin–streptomycin (Thermo Fisher, 15140122), at 22°C, in the presence of increasing concentrations of CBG dissolved in DMSO. All conditions contained 0.5% DMSO. Secondary plot analysis calculated the rate of exponential growth, from 96 to 144 h, at each concentration and normalised to the no compound vehicle control (0.5% DMSO only). For development assays, wild type (WT; AX3) *D. discoideum* cells were loaded onto nitrocellulose filters (Millipore, HABP04700) and placed on absorbent filter pads (Millipore, AP1004700) at the indicated concentration of compounds at 22°C for 24 h and then imaged to record developmental phenotypes.

### 2.3 *D. discoideum* REMI screen

To identify *D. discoideum* mutants resistant to CBG, a restriction enzyme‐mediated insertional (REMI) library containing 12,247 mutants was employed, grown with 10 μM CBG that inhibits growth by >90% in WT cells, for 10 days. Resulting CBG‐resistant cells were isolated and the location of the REMI insert determined using an inverse PCR technique to isolate the flanking DNA as previously described (Chang et al., 2012; Kelly et al., 2018; Perry et al., 2019).

### 2.4 qPCR analysis of CBG‐resistant mutants

RNA was extracted from WT and mutant cells (Qiagen, 74104), and cDNA was produced (Thermo Fisher Scientific, K1622). cDNA was then analysed by qPCR using primers 100 bp apart from the gene of interest (IPMK) and a house‐keeping gene—positive control (Ig7, DDB_G0294034). The ∆∆‐Ct method was used to calculate fold change.

### 2.5 Creation of IPMK‐RFP and hIPMK‐RFP cell lines and imaging

The *D. discoideum* IPMK gene (DDB_G0281737) was amplified by PCR of the *ipmk* gene cDNA and the human IPMK (hIPMK: Uniprot Q8NFU5, NCBI GeneID 55847) was synthesised with *D. discoideum* codon bias (GenScript) and both genes were cloned into the pDM324 plasmid containing a C‐terminal red fluorescent protein (RFP) tag (Veltman, Akar, Bosgraaf, & Van Haastert, 2009) to create an overexpressor construct. The vector was electroporated into WT (AX3) *D. discoideum* cells and selected by growth in the presence of geneticin (G418) at 10 μg/ml. Cells expressing the plasmids were imaged under 1% agar (Sigma, A5306).

### 2.6 Protein–ligand docking analysis

Tertiary structures of proteins (sequences obtained from dictybase.org or uniprot.org) were modelled using Phyre2 (Protein Homology/Analogy Recognition Engine V 2.0) and docking assays were carried out with SwissDock (www.swissdock.ch). The most likely ligand binding site showing the lowest ΔG (Gibbs free energy) was displayed with UCSF Chimera.

### 2.7 Higher order inositol phosphate level analysis

WT (AX3) and *IPMK* ^*+*^ cells (1 × 10^7^) were treated with vehicle solvent only control (0.2% DMSO) or 0.25 μM CBG or CBD for 1 or 24 h and provided blinded for analysis. In brief, cell pellets were dissolved in 1 M perchloric acid (40 μl) containing 3 mM EDTA, incubated on ice for 10 min, centrifuged (10 min, 20,000 *g* at 4°C) and the supernatant was neutralised with 1 M potassium carbonate (18 μl). Samples were incubated on ice for 2 h, centrifuged at 20 000 *g* and the supernatants collected. Samples were mixed with OrangeG (Sigma, O3756) and run on a 35% polyacrylamide gel for 17 h at 4°C at 600 V and 6 mA. Gels were stained with toluidine blue and then scanned with a desktop computer for image analysis with ImageStudio (LI‐COR, version 5.0).

### 2.8 Western blot analysis of mTOR activity in mouse embryonic fibroblast cells

Briefly, WT and *IPMK^−/−^* mouse embryonic fibroblasts (Maag et al., 2011) were treated with solvent control (vehicle, −) (0.5% DMSO), CBG or CBD (both at 4 μM), or PI3K inhibitor (10 μM) (Pictilisib, AbCam, ab141352) for 24 h in Dulbecco's modified Eagle medium (DMEM) (Sigma, D0819) containing 10% foetal bovine serum (FBS) (Thermo Fisher, 26140087). Cells lysed using radioimmunoprecipitation assay (RIPA) buffer (Thermo Fisher, 89900) containing 2% phosphatase inhibitor (1% Phosphatase Inhibitor Cocktail 3, Sigma, P0044; 1% Phosphatase Inhibitor Cocktail 2, Sigma, P5726) and 1% protease inhibitor (Thermo Fisher, 87786). Cell lysates were separated by SDS‐PAGE gel electrophoresis, transferred to PVDF membrane (Fisher, 88520) and probed with antibodies (p4EBP1; Cell Signaling Technology Cat# 9459, RRID:AB_330985: total 4EBP1, Cell Signaling Technology Cat# 4923, RRID:AB_659944: actin loading control, Sigma‐Aldrich Cat# A1978, RRID:AB_476692). Signals were detected with chemifluorescent substrate (LI‐COR, 928‐30005) and recorded on an Odyssey CLx (LI‐COR). All immuno‐related procedures involved comply with the editorial on immunoblotting and immunohistochemistry (Alexander et al., 2018).

### 2.9 Primary PBMC collection from human subjects

Healthy individuals and pwMS attending clinics at Beaumont Hospital, Dublin, Ireland, were recruited for this study. Written informed consent was obtained from each participant and the study received ethical approval from the Beaumont Hospital Ethics (Medical Research) and the Faculty of Health Sciences Research Ethics Committee, Trinity College Dublin, Ireland. The recruitment of pwMS into the study was via a consultant neurologist and all pwMS had a relapsing–remitting (RR) form of MS as defined by the revised McDonald criteria (Polman et al., 2011) including patient history, clinical signs and symptoms, physical examination and adjunctive diagnostic tools including MRI. Some pwMS were on immunomodulatory treatment including gilenya, dimethyl fumarate and plegridy. Healthy individuals with no history of autoimmune, cardiovascular, respiratory or degenerative disease were included and matched on the basis of age and gender. Details of participant demographics are presented in Table 1. PBMCs were prepared from venous whole blood samples by way of venipuncture (max 50 ml per donor collected in EDTA tubes) from healthy control participants (mean age 42.0 ± 2.6 years; *n* = 6) and pwMS (mean age 35.3 ± 5.0 years; *n* = 6). PBMCs were isolated by density separation over Lymphoprep™ (Axis‐Shield, Norway). PBMCs were (1 × 10^6^ cells/ml; 3 ml per well) maintained in culture for at least 2 h prior to treatment with rapamycin (200 nM, Sigma) and plant‐derived, highly purified CBD or CBG (100 nM) or THC:CBD combinations (20 nM:17 nM) (GW Research Ltd, Cambridge, UK) for 24 h and provided blinded for analysis.

**TABLE 1:** Demographic breakdown for peripheral blood mononuclear cell donation origin of healthy individuals (controls; HC) and pwMS

Demographic data of participants	HC	pwMS
*n*	6	6
Sex (F/M)	4/2	4/2
Age (year; mean ± SEM)	42.0 ± 2.6	35.3 ± 5.0
Disease duration (months)	n/a	49.4 ± 20.4
Medication in pwMS group		
Gilenya		1
Glatiramer acetate		1
Plegridy		1
None		2
Not reported		1

*Note*. Data are expressed as mean ± SEM.

Abbreviations: HC, healthy control; n/a, not available; pwMS, people with multiple sclerosis.

### 2.10 Western blot analysis of mTOR activity in primary PBMCs

PBMCs were lysed using RIPA buffer (Thermo Fisher, 89900) containing 2% phosphatase inhibitor (1% Phosphatase Inhibitor Cocktail 3, Sigma, P0044; 1% Phosphatase Inhibitor Cocktail 2, Sigma, P5726) and 1% protease inhibitor (Thermo Fisher, 87786). Cell lysates were separated by SDS‐PAGE gel electrophoresis, transferred to a PVDF membrane (Fisher, 88520) and probed overnight with antibody for p4EBP1 (Cell Signalling Technology, 9459), total 4EBP1 (NEB, 4923) and actin (Sigma, A1978). Signals were detected with chemifluorescent substrate (LI‐COR, 928‐30005) and recorded on an Odyssey CLx (LI‐COR).

### 2.11 Data and statistical analysis

To ensure unbiased analysis when comparing different treatments, all samples were randomly assigned to the treatments and measures of all test groups were balanced within each assay. Data and statistical analysis comply with the recommendations on experimental design and analysis in pharmacology (Curtis et al., 2018). Time course and dose as a repeated measure was analysed by two‐way ANOVA in Figures 2a and 3d. Normality tests for data were carried out using Shapiro–Wilk's test. If data were found to be normally distributed, then a one‐way ANOVA with a post‐test of either Dunnett's or Bonferroni's multiple comparisons tests was carried out, as specified. The post hoc tests were conducted only if F in ANOVA achieved *P* < .05 and there was no significant variance inhomogeneity. If data were found to not be normally distributed, then a Kruskal–Wallis test with a Dunn's multiple comparison post‐test or Mann–Whitney test was carried out to test for significance. Sample sizes subjected to statistical analysis consisted of at least 5 independent experiments per group (*n* = 5). Significance was found if *p* ≤ 0.05. Data are represented as mean ± SEM.

### 2.12 Materials

CBD (>95%), CBG (>95%) and THC (94.5%) were provided by GW Research Ltd. Details of other materials and suppliers are provided in specific sections.

### 2.13 Nomenclature of targets and ligands

Key protein targets and ligands in this article are hyperlinked to corresponding entries in the IUPHAR/BPS Guide to PHARMACOLOGY http://www.guidetopharmacology.org and are permanently archived in the Concise Guide to PHARMACOLOGY 2019/20 (Alexander et al., 2019).

## 3 RESULTS

### 3.1 CBD and CBG increase mTOR activity in WT *D. discoideum* cells

**FIGURE 1:**
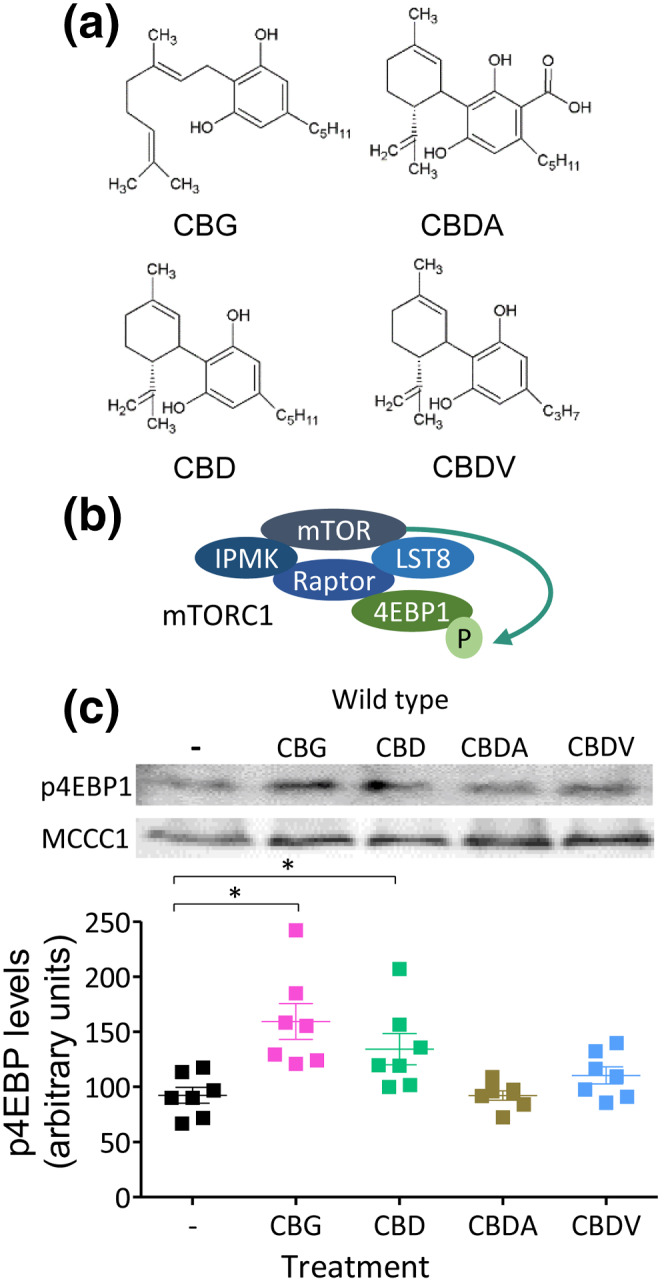
Assessing the effect of structurally distinct phytocannabinoids on mTORC1 activity in *D. discoideum* cells. (a) Structures of abundant phytocannabinoids found in cannabis, including CBG, CBD, CBDA and CBDV. (b) The mTOR complex 1 (mTORC1) is composed of four subunits including IPMK, raptor, Lst8 and mTOR and functions to phosphorylate the eukaryotic translation initiation factor 4E‐binding protein 1 (4EBP1), providing a means to monitor mTORC1 activity, following phytocannabinoid treatment. (c) Treating *D. discoideum* WT cells with CBG, CBD, CBDA or CBDV (0.25 μM for 1 h) or solvent only control (−), enabled analysis of mTORC1 activity by western blot using an antibody against phosphorylated 4EBP1 (p4EBP1; *n* = 7) with loading control (MCCC1). Data were normally distributed as tested by Shapiro–Wilk's test and significance assessed by one‐way ANOVA with Dunnett's post‐hoc, *p ≤ 0.05; DF:4,30, F = 7.0 with treatment against control. Graph shows mean ± SEM

We initially investigated the effect of CBD, CBG, CBDA and CBDV on mTORC1 signalling in *D. discoideum* (Figure 1a,b). We employed a western blot approach, using an antibody against the phosphorylated form of the eukaryotic translation initiation factor 4E‐binding protein 1 (4EBP1) as a direct target of mTORC1 in *D. discoideum* (Chang et al., 2020; Nichols et al., 2020), that is reduced in starvation and following treatment with two established mTOR inhibitors (Warren et al., 2020) (Figure 1b). Cells were treated for 1 h with each phytocannabinoid at 0.25 μM, a concentration similar to that reported in rodent brain tissue following oral dosing of CBD and in human plasma samples from clinical trials with Epidiolex® (Devinsky et al., 2018), and were analysed for phospho‐4EBP1 (p4EBP1) levels (Figure 1c). CBD treatment significantly increased 4EBP1 phosphorylation by approximately 45% compared with vehicle control. CBG promoted a similar increase in mTORC1 activation, however the structurally similar CBDA and CBDV did not significantly alter mTORC1 activity. We then investigated the mechanisms behind this effect in *D. discoideum*, initially focusing on CBG as a potential new therapeutic compound.

### 3.2 CBG inhibits *D. discoideum* growth but not development

**FIGURE 2:**
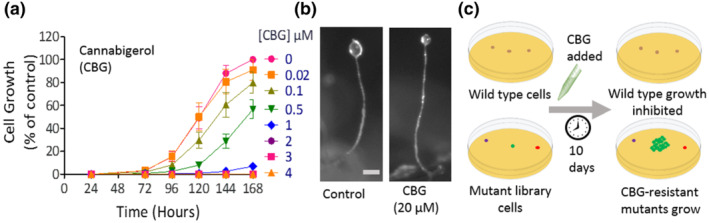
CBG inhibits growth of *D. discoideum* WT cells but not development, enabling a screen to identify CBG‐resistant mutants. (a) WT cells were grown in the presence of increasing concentrations of CBG (and solvent‐only control; 0 μM) for 1 week. CBG inhibited cell growth in a concentration‐dependent manner (*n* = 10). Growth was presented as a cell number normalised to solvent‐only control. Data, analysed by two‐factor ANOVA, showed a significant reduction relating to CBG (*p ≤ 0.05). Graph shows mean ± SEM. (b) WT *D. discoideum* cells, developed for 24 h under starvation conditions to form fruiting bodies, comprising a rounded spore head held aloft by a stalk (scale bar = 100 μm). In the presence of CBG (20 μM), development of the fruiting body was unaffected. (c) Schematic representation of a mutant library screen to identify mutants resistant to the effect of CBG, where WT cell growth is inhibited but individual mutants resistant to the cellular effects of CBG continue to grow, allowing these resistant mutants to be isolated and genes controlling CBG sensitivity to be identified

To investigate the mechanism of action of CBG on mTORC1 signalling, we first analysed the effect of CBG on both cellular growth and multicellular development in *D. discoideum*. CBG caused a concentration‐dependent reduction in growth, from a small reduction at 0.1 μM to total inhibition of growth at ≥2 μM (Figure 2a). Because in this model, starvation induces a developmental cycle where cells employ a host of proteins and signalling pathways that are distinct to those necessary for growth (Schaf et al., 2019), we also investigated the effects of CBG on this process. Under control conditions, starvation induced the development of mature fruiting bodies comprising a round spore head held above the substratum by an elongated stalk and a basal disc (Figures 2b and S2). CBG treatment (up to 20 μM) had no effect on this developmental process, allowing phenotypically normal fruiting body formation (Figure 2b). These results suggest that proteins and signalling pathways necessary for growth, but not development, may be targeted by CBG and that this effect is not through a generalised toxic mechanism leading to cell death, because cells survive and develop into mature fruiting bodies in the presence of high concentrations of CBG.

### 3.3 Inositol polyphosphate multikinase (IPMK) regulates the effect of CBG on *D. discoideum* cellular growth and mTOR activity

Pharmacogenetics studies in *D. discoideum* often involve screening an insertional mutant library to isolate cells showing reduced sensitivity to compounds (Figure 2c) and identification of the gene(s) affected in these cells provides valuable insight into the signalling mechanism affected by the compound (Chang et al., 2012; Kelly et al., 2018; Perry et al., 2019; Schaf et al., 2019). Thus, we performed a screen using a pool of 12,247 individual mutants, to isolate cells resistant to the effect of CBG on growth (Figure 2c), enabling the identification of proteins controlling CBG sensitivity.

**FIGURE 3:**
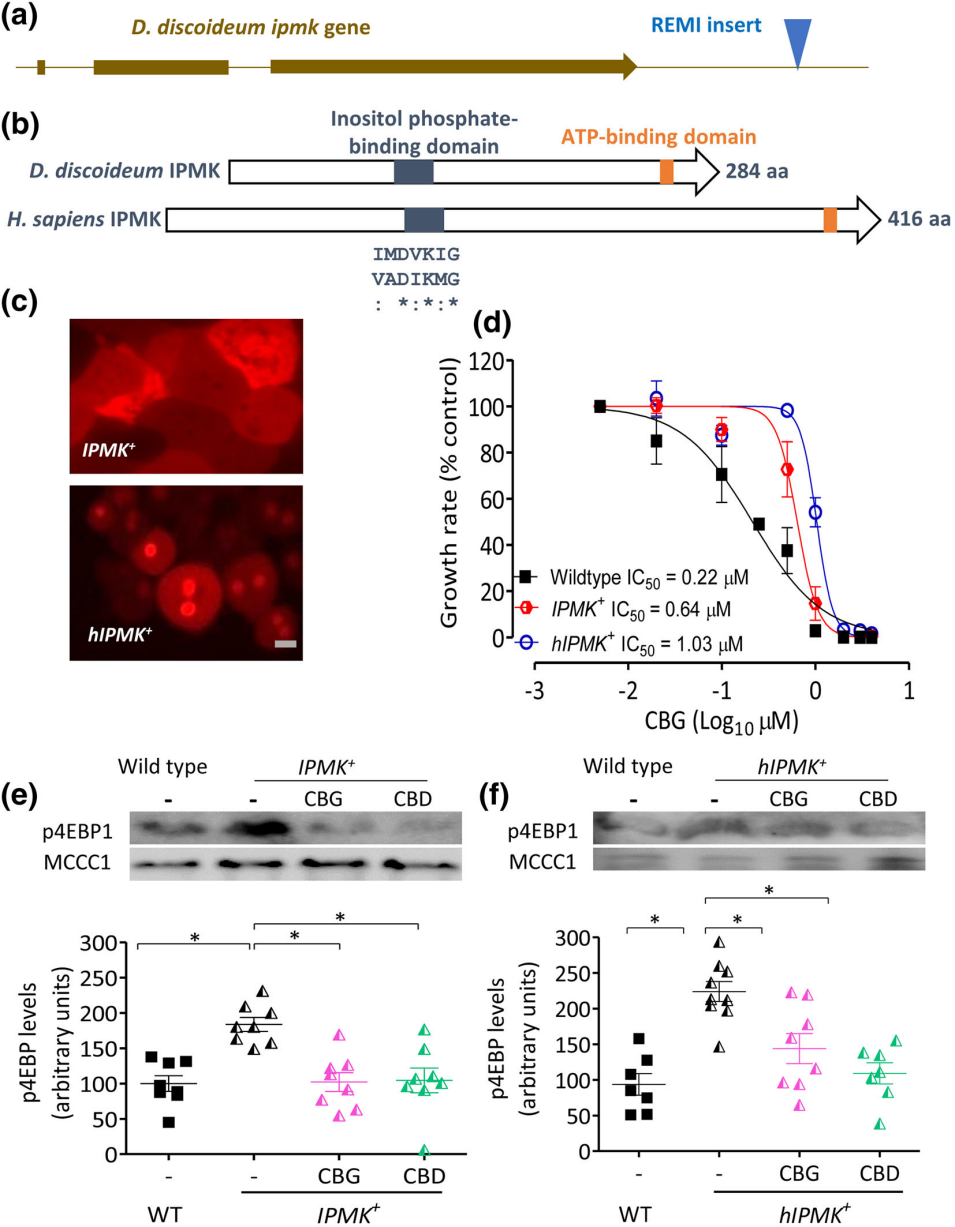
Elevated expression of *D. discoideum* or human IPMK regulates the effect of CBG and CBD on growth and mTORC1 activity in *D. discoideum*. (a) A *D. discoideum* insertional mutant showing resistance to CBG contained a mutagenic (REMI) insert 885 bp downstream from the *ipmk* open‐reading frame (*DDB_G0281737*; Uniprot Q54TI0). (b) The *D. discoideum* IPMK protein shares 33% identity to the human protein (hIPMK; Uniprot Q8NFU5) and both contain inositol phosphate‐ and ATP‐binding domains (highlighted; similar aa residues ‘:’, identical aa ‘*’). (c) Fluorescent images of *D. discoideum* cells expressing the *D. discoideum* IPMK‐RFP (*IPMK* ^*+*^) protein localised to the cytoplasm and the human hIPMK‐RFP (*hIPMK* ^*+*^) localised to the nucleus and cytoplasm (bar = 3 μm). (d) Growth sensitivity to CBG, analysed by two‐factor ANOVA, showed a significantly reduction in *IPMK* ^*+*^ (*n* = 6) and *hIPMK* ^*+*^ (*n* = 7) compared with WT cells (*n* = 10) (*p* ≤ 0.01 for both) (*p ≤ 0.05). (e, f) mTORC1 activity in *IPMK* ^*+*^ and *hIPMK* ^*+*^ was assessed following treated with either solvent (−), CBG or CBD (0.25 μM) for 1 h by monitoring phosphorylation of 4EBP1 by western blot (*n* = 8), with loading control (MCCC1). (e) In the absence of phytocannabinoids, *IPMK* ^*+*^ cells had significantly higher mTORC1 activity than WT cells and both phytocannabinoids significantly reduced mTORC1 activity. (f) In the absence of phytocannabinoids, *hIPMK* ^*+*^ cells had significantly higher mTORC1 activity than WT cells and both phytocannabinoids significantly reduced mTORC1 activity. Data were normally distributed with Shapiro–Wilk's test and a one‐way ANOVA with Bonferroni's multiple comparison test was carried out to test significance, *p ≤ 0.05. Graphs show mean ± SEM and significance was tested against WT control and *IPMK* ^*+*^ or *hIPMK* ^*+*^ control

Through the use of a CBG resistance screen, we identified one mutant, in three independent experiments containing the mutagenic DNA cassette inserted 885 bp downstream from the stop codon of the gene encoding IPMK (*DDB_G0281737*; *ipmk*) (Figure 3a). The encoded 284 aa protein (IPMK, Uniprot Q54TI0) contains conserved inositol phosphate‐binding and ATP‐binding domains (Figures 3b and S3), consistent with the orthologous human protein (hIPMK; Uniprot Q8NFU5). Because this insertion site was not within the open reading frame of the gene and was thus unlikely to disrupt protein translation, we investigated changes in *ipmk* transcriptional levels in this mutant using real‐time PCR analysis. This analysis demonstrated a significant threefold increase in *ipmk* expression in the CBG‐resistant mutant compared with WT cells (Figure S4A), suggesting that elevated IPMK expression provides resistance to the cellular effect of CBG on *D. discoideum* growth.

We continued the analysis of a role for IPMK in regulating the cellular effects of CBG by overexpressing fluorescently tagged versions of the *D. discoideum* and *Homo sapiens* proteins. Both the *D. discoideum* IPMK (in *IPMK* ^*+*^) and the human IPMK protein (in *hIPMK* ^*+*^) were expressed with a C‐terminal RFP tag (Figure S4B). Fluorescent imaging of the resulting cells indicated that IPMK‐RFP was localised in the cytosol, whereas hIPMK‐RFP was localised in the cytoplasm and nucleus (Figure 3c), consistent with the localisations defined in mammalian studies (Nalaskowski, Deschermeier, Fanick, & Mayr, 2002). CBG resistance during growth was then compared between WT, *IPMK* ^*+*^ and *hIPMK* ^*+*^ cell lines, visualised in a secondary plot showing the rate of exponential growth at different CBG concentrations for each cell line (Figures 3d and S5). These analyses indicated CBG IC_50_ values for WT of 0.22 μM, whereas *IPMK* ^*+*^ cells showed a threefold increase to 0.64 μM and *hIPMK* ^*+*^ cells showed a fivefold increase to 1.03 μM. These data confirm that elevated *D. discoideum* IPMK levels increase resistance to the effect of CBG on growth over WT cells and this cellular role was conserved by the expression of the human IPMK protein.

To investigate a potential direct mechanism for both phytocannabinoids against the *D. discoideum* and human IPMK proteins, in silico molecular docking analysis was employed. This analysis suggested a common binding site for both CBG and CBD on the *D. discoideum* IPMK (at Glu148) (Figure S6), but this binding site was not consistent with that shown for the human protein. Thus, it remains unclear if this mechanism is through direct inhibition of both IPMK enzymes or through a secondary target.

Because overexpression of IPMK increased resistance to the effect of CBG on cellular growth, we also examined changes in mTORC1 activity in these cells, using phosphorylation of 4EBP1 as a readout (Chang et al., 2020). Elevated expression of IPMK promoted a significant, 1.8‐fold increase in mTORC1 activity as compared with WT cells (Figure 3e), consistent with a role for elevated levels of IPMK enhancing mTORC1 activity. However, in contrast to that observed in WT cells, CBG or CBD treatment (0.25 μM for 1 h) of *IPMK* ^*+*^ cells caused a decrease in the activity of mTORC1, returning the elevated p4EBP1 levels to that comparable with WT vehicle‐treated cells (Figure 3e). Furthermore, expression of the human IPMK protein caused a significant 2.2‐fold increase in mTORC1 activity as compared with WT cells (Figure 3f), consistent with overexpression of the native IPMK protein. In addition, CBG or CBD treatment (0.25 μM for 1 h) of *hIPMK* ^*+*^ cells promoted a decrease in the activity of mTORC1, returning the elevated p4EBP1 levels to that comparable with WT vehicle‐treated cells (Figure 3f). However, this outcome suggests differential effects of CBG and CBD on mTORC1 activity, where in WT cells, these phytocannabinoids function to activate mTORC1 signalling, whereas they reduce mTORC1 signalling following IPMK overexpression, suggesting that these phytocannabinoids provide an IPMK‐dependent effect on this signalling pathway.

### 3.4 IPMK regulates the effect of CBG via a PI3K/PKB‐dependent mechanism in *D. discoideum*

**FIGURE 4:**
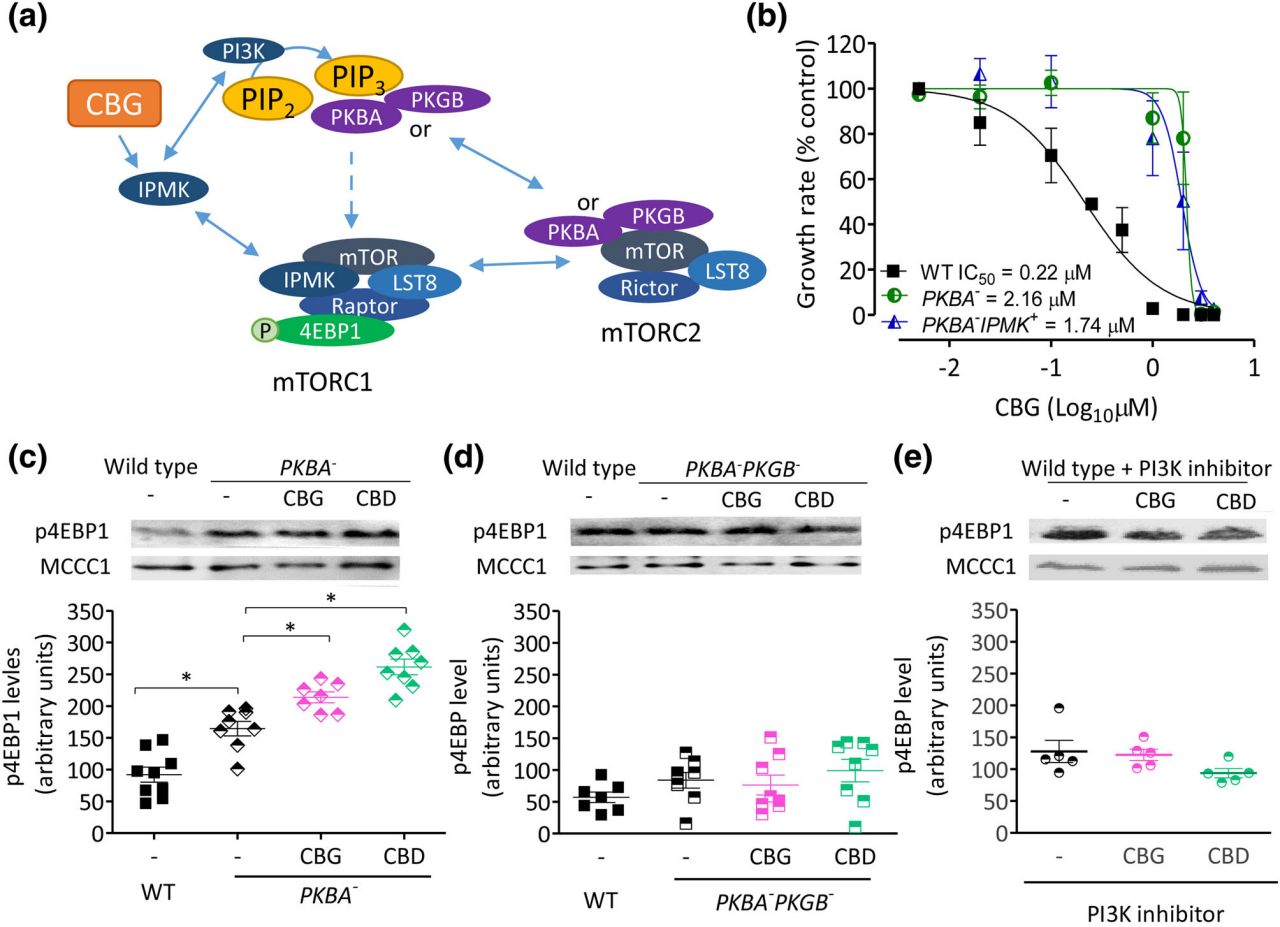
Phytocannabinoid effects on cell growth and mTORC1 activation are dependent upon PKB and PI3K activity in *D. discoideum*. (a) Regulation of mTORC1 activity occurs through a complex pathway, including PI3K and PKB (in *D. discoideum*: PKBA and PKGB), and with multiple components shared between mTORC1 and mTORC2 complexes. (b) Assessing CBG‐dependent growth sensitivity following loss of PKBA (in *PKBA* ^*−*^; *n* = 7) and following overexpression of IPMK (*PKBA* ^*−*^: *IPMK* ^*+*^; *n* = 5) showed that ablation of PKB and additional overexpression of IPMK resulted in significantly elevated IC_50_ compared with WT cells (*n* = 10) (*p* ≤ 0.05). Data were not normally distributed with Shapiro–Wilk's test; therefore, a Kruskal–Wallis test with Dunn's multiple comparison post‐test was carried out to determine significance; Kruskal–Wallis statistic = 10.59. Graphs show mean ± SEM and significance was tested against WT control and *PKBA* ^*−*^ or *PKBA* ^*−*^ *PKGB* ^*‐*^. (c–e) To assess mTORC1 activity, cells were treated with either solvent (−) or CBG or CBD (0.25 μM) for 1 h and cells were analysed by western blot for phosphorylation of 4EBP1, with loading control (MCCC1), in (c) the absence of PKBA, in (d) the absence of PKBA and PKGB activity and in (e) the absence of PI3K activity, where WT cells were treated with a specific PI3K inhibitor (LY294002: 60 μM). (c, d) Data were normally distributed with Shapiro–Wilk's test and significance provided by one‐way ANOVA with Bonferroni's multiple comparison test. (c) *PKBA* ^−^ cells had a significantly higher level of p4EBP1 than WT cells and both CBG and CBD caused a significant increased level of p4EBP1 compared with vehicle treatment (*n* = 8), *p ≤ 0.05; DF:3,28, F = 43.1. (d) *PKBA* ^*−*^ *PKGB* ^−^ cells showed no change in level of p4EBP1 (*n* = 8); DF:3,27, F = 1.4. (e) In the presence of PI3K inhibitor, cells showed no change in level of p4EBP1 (*n* = 5). The data were not normally distributed according to the Shapiro–Wilk's test and no significance was found by the Kruskal–Wallis test with Dunn's multiple comparison post‐test

Because mTORC1 activity may be regulated by the gain of components shared with and thus sequestered by the mTORC2 complex, such as PKB (Figure 4a), we investigated the effect of CBG in the absence of PKB. In these experiments, we initially focused on the *PKBA* ^−^ cell line, a mutant lacking the most characterised PKB isoform (Tang et al., 2011) and examined these cells for growth sensitivity to CBG. Loss of this enzyme increased the IC_50_ for CBG 10‐fold, from 0.22 μM in WT cells to 2.16 μM in the mutant, clearly supporting a role for PKBA in the CBG‐dependent interruption of cellular function during growth (Figures 4b and S7). This effect was not rescued by elevating expression of IPMK in these cells (to produce *PKBA* ^*−*^ *IPMK* ^*+*^ cells), where resulting cells displayed a ninefold increase in the IC_50_ for CBG to 1.94 μM (Figure 4b). The large decrease in CBG sensitivity following loss of PKBA is consistent with a cellular mechanism of the compound that is dependent upon PKBA activity but does not differentiate between effects of CBG through PI3K/PKBA signalling or through reducing mTORC2 formation or via an effect on IPMK to regulate mTORC1 signalling that is dependent upon upstream PI3K/PKBA signalling.

To investigate a role for PKB activity in CBG‐ and CBD‐dependent mTORC1 regulation, we analysed mTORC1 activity in *PKBA* ^−^ cells following treatment with CBG and CBD, assessing mTORC1 activity using the previous assay (phosphorylated 4EBP1 levels, with 0.25‐μM phytocannabinoid, 1‐h treatment). In the absence of phytocannabinoid, loss of PKBA significantly increased mTORC1 activity by around twofold above WT levels (Figure 4c), potentially due to a block in formation of mTORC2, thereby increasing mTORC1 formation and thus activity (Figure 4a). In *PKBA* ^−^ cells, both CBG and CBD treatment still increased mTORC1 activity by 1.4‐ or 1.5‐fold, respectively. However, because *D. discoideum* contains two PKB proteins (PKBA and PKGB; DDB_G0290157), we also analysed mTORC1 activity and response to CBG and CBD treatment in cells lacking both proteins (*PKBA* ^*−*^ *PKGB* ^−^). Here, we found that loss of both PKB proteins did not elevate mTORC1 activity above WT levels and that phytocannabinoid effects on mTORC1 activation were lost (Figure 4d), suggesting that PKB signalling is necessary for cannabinoid‐dependent mTORC1 activation. To extend this result, we also employed a pharmacological approach to block PI3K activity (Figure 4e), as the key activator of PKB function, where inhibition of PI3K also blocked the effect of CBG and CBD on 4EBP1 phosphorylation. Finally, we show that these effects are unlikely to occur via mTORC2, because phosphorylation of Ras guanine nucleotide exchange factor S (GefS), a *D. discoideum* guanine nucleotide exchange factor targeted by mTORC2 (Kamimura et al., 2008), is not downregulated by either cannabinoid (Figure S8). These data suggest that PI3K/PKB signalling must be active for the CBG‐ and CBD‐dependent activation of mTORC1 signalling but do not differentiate between IPMK‐dependent and PI3K/PKB‐dependent mechanisms.

### 3.5 CBG and CBD elevate IPMK‐dependent inositol phosphate phosphorylation activity in *D. discoideum*

**FIGURE 5:**
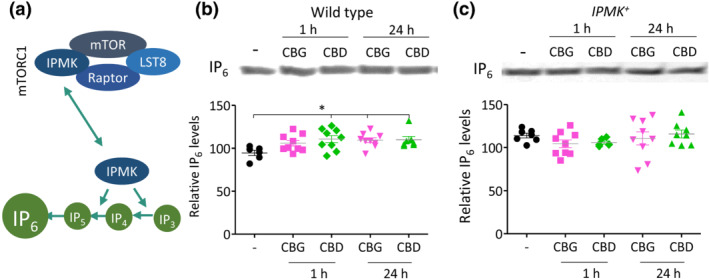
Phytocannabinoids upregulate the inositol phosphate catalytic activity of IPMK in *D. discoideum*. (a) IPMK has a catalytic role in the phosphorylation of higher order inositol phosphates, to produce IP_4_ and IP_5_ that are essential for the creation of IP_6_. (b, c) Changes in IP_6_ levels were measured in the presence of CBG and CBD in WT and *IPMK* ^+^ *D. discoideum* cells exposed to CBG or CBD (0.25 μM) or vehicle (−) control for 1 or 24 h followed by inositol phosphate extraction and quantification. Data were not normally distributed (Shapiro–Wilk's test); therefore, a Kruskal–Wallis test with Dunn's multiple comparison post‐test was used to evaluate significance. Graphs show mean ± SEM. (b) In WT cells, CBD treatment significantly elevated IP_6_ levels compared with control following 1‐ and 24‐h treatment (*p* ≤ 0.05) and CBG significantly elevated IP_6_ levels following 24‐h treatment (*p* ≤ 0.05) compared with control (*n* = 6–9); Kruskal–Wallis statistic = 10.8. (c) In *IPMK* ^+^, no significant effects were found of phytocannabinoid treatment on IP_6_ levels (*n* = 7–9); Kruskal–Wallis statistic = 4.3

To distinguish between a CBG‐ and CBD‐dependent effect on IPMK or PI3K/PKB signalling and because IPMK contributes to the synthesis of higher order inositol phosphates such as IP_4–6_ (Figure 5a), we assessed the effects of CBG and CBD on enhancing production of higher order inositol phosphates (Maag et al., 2011). In these experiments, WT and *IPMK* ^*+*^ cells were treated with CBG or CBD (0.25 μM for 1 and 24 h) (Figure 5b), reflecting a rapid (metabolic) response and a long‐term (transcriptional/translational) response, respectively, inositol phosphates were extracted and separated using polyacrylamide gel electrophoresis and IP_6_ levels were quantified. Here, CBD significantly increased IP_6_ levels following acute (1 h) exposure (15% increase) and both CBG and CBD significantly increased IP_6_ levels following chronic (24 h) exposure (15% increase). This increase is likely to be highly relevant to cell function because a previously reported 18% increase in IP_6_ levels modulates calcium influx over the plasma membrane to serve as a cell signal response (Larsson et al., 1997). Repeating this analysis in cells overexpressing IPMK (Figure 5c) shows that these CBG‐ and CBD‐regulated effects are lost, consistent with that shown for mTORC1 activation. These data suggest a role for CBG and CBD in activating IPMK to elevate production of higher order inositol phosphates.

### 3.6 IPMK regulates the effect of CBG and CBD on mTORC1 activation in MEF cells

**FIGURE 6:**
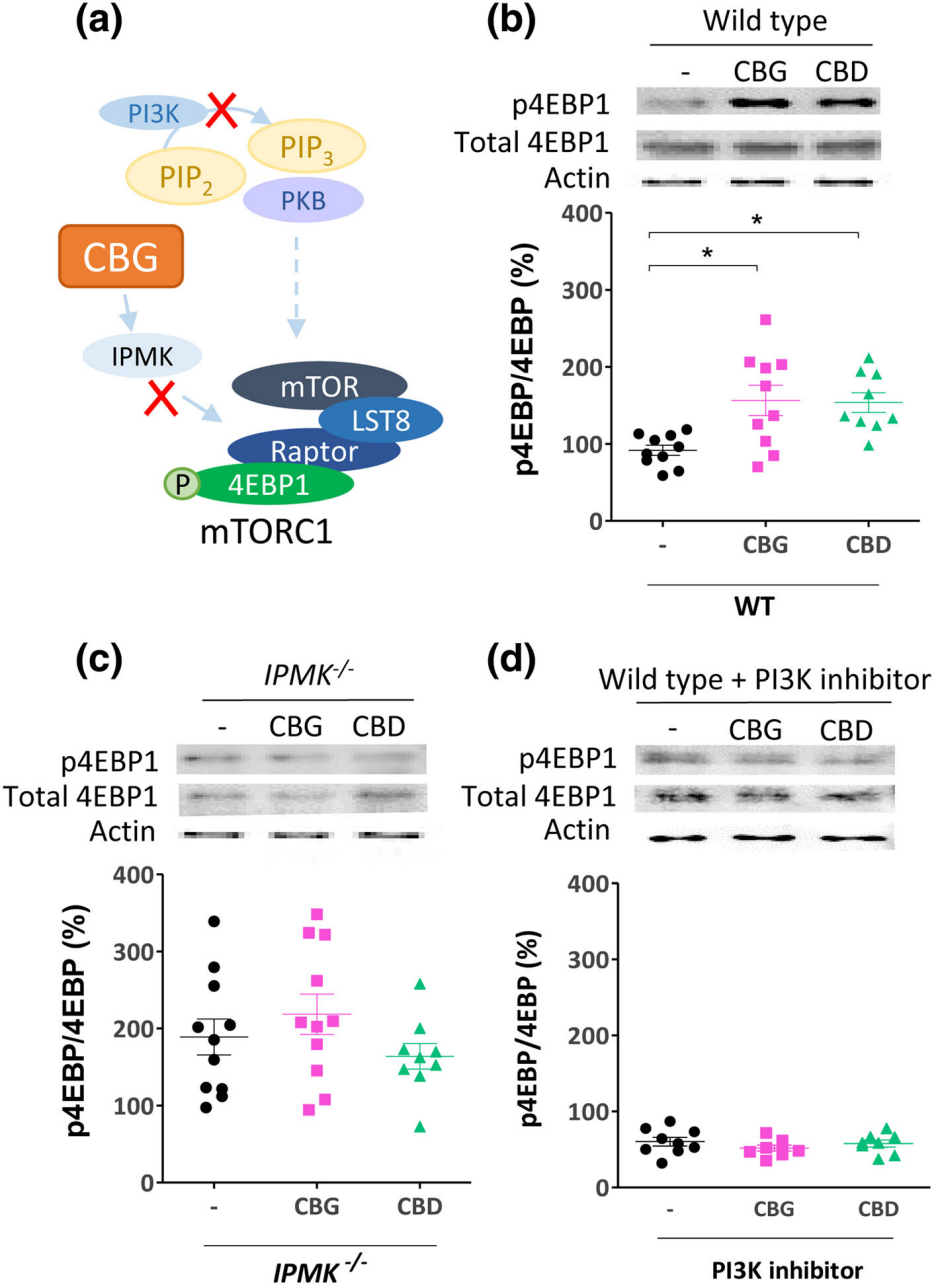
Phytocannabinoids upregulate mTORC1 activation in mouse embryonic fibroblasts through IPMK and PI3K‐dependent signalling. (a) Analysing a role for CBG and CBD in mTORC1 signalling through IPMK and PI3K/PKB signalling in mouse embryonic fibroblasts employed *IPMK^−/−^* cells and pharmacological inhibition of PI3K/PKB signalling (10 μM Pictilisib). (b–d) The effect of 24 h treatment with 4 μM CBG or CBD on mTORC1 activity in MEF cells was measured by western blot analysis to measure p4EBP1 as a proportion of total 4EBP1 in each treatment (see also Figure S9), with actin as control. Data were normally distributed with Shapiro–Wilk's test and a one‐way ANOVA with Dunnett's multiple comparison test was used to test significance of treatment against controls. Graphs show mean ± SEM. (b) In WT MEF cells, CBG and CBD treatment significantly increased the proportion of p4EBP1 levels (*n* = 9) (*p ≤ 0.05); DF:2,26, F = 6.8. (c) CBD and CBG treatment of MEF cells lacking IPMK (*IPMK* ^*−/−*^) did not affect the proportion p4EBP1 levels (*n* = 9); DF:2,28, F = 1.4. (d) CBD and CBG treatment of MEF cells lacking PI3K activity (treated with a PI3K inhibitor; 10 μM Pictilisib) did not affect the proportion p4EBP1 levels (*n* = 9); DF:2,22, F = 0.8

Because we have proposed a mechanism for CBG and CBD in the regulation of mTORC1 signalling dependent upon IPMK in *D. discoideum* (Figure 6a), we aimed to translate this effect into mammalian cells. Here, we employed MEF cells, treated with CBG or CBD for 24 h, at 4 μM, a concentration used in a variety of *in vitro* experiments (Figure 6b) (Kozela et al., 2011; O'Sullivan, Sun, Bennett, Randall, & Kendall, 2009), but chosen to ensure strong pathway activation and analysed mTORC1 activity. In WT MEF cells, CBG and CBD treatment induced a significant increase in p4EBP1 following treatment with either phytocannabinoid, consistent with that shown in *D. discoideum* (Figures 6b and S9). To confirm that these effects were dependent upon IPMK activity, we repeated this analysis using an MEF cell line lacking the IPMK protein (Kim et al., 2011), which showed no change in p4EBP1 levels following treatment (Figure 6c), confirming phytocannabinoid‐dependent effects on mTORC1 activation through IPMK. We also examined the PI3K dependency of this phytocannabinoid effect, by treating cells with CBG or CBD following pharmacological inhibition of PI3K activity, where no phytocannabinoid‐dependent increase in mTORC1 activation was observed (Figure 6d). These results confirm and extend those found in *D. discoideum*, suggesting that both CBG and CBD act to increase mTORC1 activity through enhancing IPMK activity in MEF cells and are dependent on a functional PI3K signalling pathway.

### 3.7 CBG, CBD and a mixture of THC and CBD differentially regulate mTOR activation in primary PBMCs derived from pwMS

**FIGURE 7:**
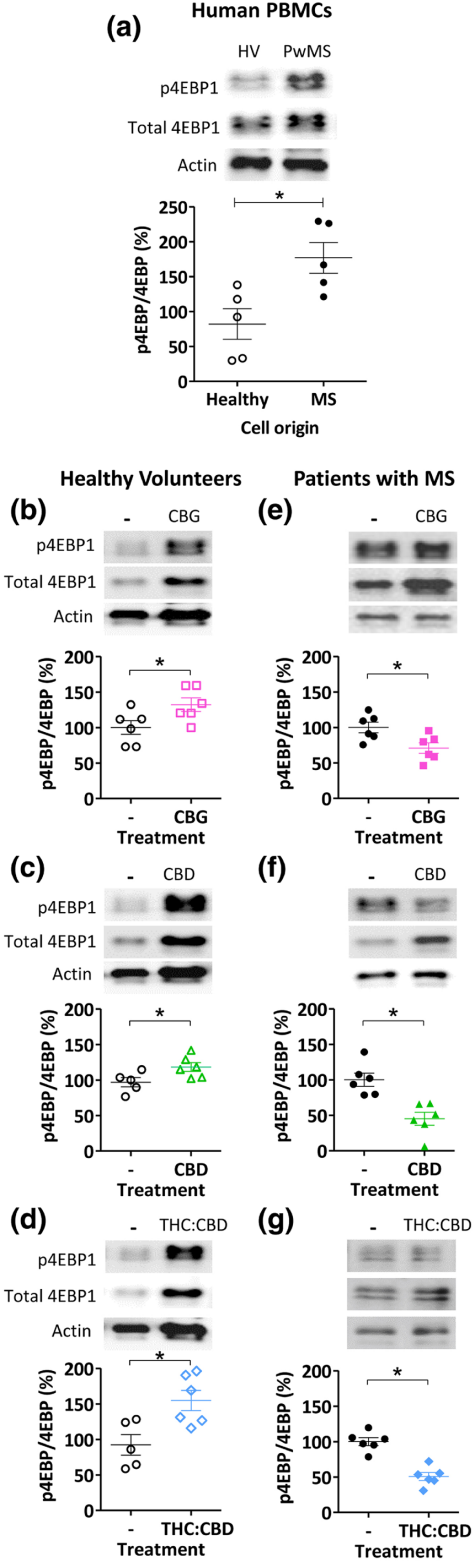
Phytocannabinoids differentially regulate mTORC1 activity in primary human peripheral blood mononuclear cells (PBMCs) from healthy individuals and pwMS. (a) To examine the therapeutic relevance of mTORC1 activity as a target for CBD and CBG, p4EBP1 levels as a proportion of total 4EBP1 were measured in each treatment, with actin as control, in PBMCs derived from healthy individuals and pwMS. PBMCs isolated from pwMS were found to have a significantly higher ratio of phosphorylated 4EBP1 to total 4EBP1. (b–d) mTORC1 activity in PBMCs isolated from healthy individuals following 24‐h treatment with CBG (100 nM) or CBD (100 nM) or a combination of THC and CBD (20 nM:17 nM, respectively) was measured by western blot analysis using p4EBP1 as a proportion of total 4EBP1 in each treatment, using actin as control, showing significant increase in mTORC1 activity following phytocannabinoid treatment (*n* = 6). (e–g) A similar analysis of PBMC cells originating from pwMS showing significant decrease in mTORC1 activity following phytocannabinoid treatment (*n* = 6). (a–i) Data were not distributed according to Shapiro–Wilk's test; therefore, a Mann–Whitney test was carried out to test for significance, *p ≤ 0.05. Graphs show mean ± SEM

We continued by investigating an effect of CBG and CBD in regulating mTOR activity in a clinical setting, using PBMCs from healthy individuals and pwMS. Cells were treated with rapamycin (200 nM), CBG (100 nM), CBD (100 nM) (to parallel concentrations used in *in vitro* assessments elsewhere and in approximate range to the clinical concentrations found in people with MS during treatment; Stott, White, Wright, Wilbraham, & Guy, 2013) and the combination THC:CBD (20:17 nM, in the range of clinical plasma concentrations; Leussink et al., 2012; Stott et al., 2013) or solvent‐only control for 24 h and analysed for mTORC1 activity. In these experiments, separate blots were used for each antibody, to ensure no cross‐over between total and phoshpo‐4EPB1 readouts or loss of protein due to membrane stripping. Cells derived from pwMS were found to have an increased ratio of p4EBP1:4EBP1 compared with cells from matched healthy controls, consistent with upregulation of this pathway in the disease state (Figure 7a). Treatment of cells with rapamycin reduced the proportion of p4EBP1:4EBP1 in both healthy matched controls and pwMS (Figure S10). Treatment of cells derived from healthy individuals with CBG, CBD or THC:CBD increased the p4EBP1:4EBP1 ratio by 1.3‐, 1.2‐ and 1.5‐fold, respectively (Figure 7b–d). In contrast, treatment of cells derived from pwMS with CBG, CBD and THC:CBD caused a decrease in the p4EBP1:4EBP1 ratio of 1.4‐, 2.2‐ and 1.8‐fold, respectively (Figure 7e–g). These results confirm a role for CBG, CBD and THC:CBD in mTORC1 regulation in human PBMCs dependent upon disease state.

## 4 DISCUSSION

Phytocannabinoids have demonstrated efficacy in preclinical models of MS (Feliu et al., 2015; Giacoppo et al., 2017) and in clinical studies relating to spasticity associated with MS (Etges et al., 2016; Novotna et al., 2011). In addition, CBD has been shown to be effective in preclinical models of psychosis (Renard et al., 2016; Renard, Norris, Rushlow, & Laviolette, 2017), breast cancer (Sultan et al., 2018) and tuberous sclerosis complex (Serra et al., 2019). One suggested mechanism for CBD treatment of epilepsy and cancer is through the downregulation of hyperactivated mTORC1 found in the disease state (Serra et al., 2019; Sultan et al., 2018). In contrast, in the spinal cord neurons of a mouse model of MS, the mTORC1 pathway has been suggested to be downregulated after induction of the disease state (Giacoppo et al., 2017), where CBD elevates mTORC1 signalling and this could improve the progression of the disease due to the arrest of myelin loss. A similar therapeutic mechanism of mTORC1 activation was also proposed for CBD in a mouse model of schizophrenia‐related psychosis (Renard et al., 2016; Renard et al., 2017) and in the anticonvulsant effect of CBD in cocaine‐induced seizures in a mouse model (Gobira et al., 2015). These conflicting studies suggest that further analysis of the effects of cannabinoids on mTORC1 signalling is necessary to explain this variance.

Our study identifies a role for CBD and CBG in regulating mTORC1 activity, where in WT *D. discoideum* cells, CBG and CBD activate mTORC1 signalling. Through an unbiased growth resistance screen, similar to that employed in an earlier report for CBD (Perry et al., 2019), we identify a role for IPMK in controlling phytocannabinoid sensitivity, where elevated expression of the *D. discoideum* protein or the orthologous human protein leads to a threefold to fivefold increase in IC_50_ value for CBG. At high CBG concentration (>1 μM), cells remain sensitive to treatment, likely representing effects on a secondary target. Interestingly, treating cells showing elevated expression of either *D. discoideum* or *H. sapiens* IPMK using a single concentration of cannabinoid reported in rodent brain tissue following oral dosing of CBD and in human plasma samples from clinical trials with Epidiolex® (Devinsky et al., 2018) inverses the effects of phytocannabinoids to cause a reduction in mTORC1 activity upon treatment, although a dose‐dependent analysis of this effect would provide further insight. We also confirm that the phytocannabinoid‐dependent regulation of mTORC1 activity is lost in mouse embryonic fibroblasts following ablation of IPMK, validating this mechanism in mammalian cells. We also demonstrate that treatment of PBMCs from healthy individuals with CBG, CBD or THC:CBD increases mTORC1 activity, whereas treatment of PBMCs from pwMS reduces mTORC1 activity, validating related effects in a preclinical setting. It is worth noting that, in this PBMC analysis, CBD may partly counteract CB_1_ receptor activation (Morales, Goya, Jagerovic, & Hernandez‐Folgado, 2016) and CB_1_ receptors may regulate mTORC1 signalling in a complex context‐dependent manner (Puighermanal et al., 2009), providing additional regulation of this important complex.

In the present study, we have employed *D. discoideum* as a 3Rs model organism to investigate a mechanism underlying the CBG‐ and CBD‐dependent activation of mTORC1 signalling. In this model, we show that CBD acutely increases mTORC1 activity at concentrations used clinically and consistent with that shown in neurons in MS and psychosis studies (Giacoppo et al., 2017; Renard et al., 2016; Renard et al., 2017). Interestingly, CBG also triggers this effect, whereas two closely related phytocannabinoids, CBDV and CBDA, do not activate this response, suggesting potential structural specificity for these phytocannabinoid in regulating mTORC1. We propose that the inverted response to these phytocannabinoids following elevated expression of IPMK may be due to the enzyme functioning as a rate limiting factor for mTORC1 activity and that these phytocannabinoids may trigger the IPMK preferential involvement in the mTORC1 complex, leading to enhanced mTORC1 activity. This model is supported by our data showing that elevated expression of IPMK in *D. discoideum* increases mTORC1 activity and loss of IPMK in MEF cells blocks phytocannabinoid‐dependent mTORC1 activation. In this model, with elevated expression of IPMK, mTORC1 activity is maintained through a reduced reliance on PI3K activity, because IPMK also shows PI3K activity (Kim, Ahn, Kim, Lee, & Kim, 2017). Phytocannabinoid treatment of these cells enhances the direct activity of IPMK, including function in the mTORC1 complex, thereby leaving cells with a reduced PI3K/PKB signalling pathway necessary for the activation of mTORC1, with an overall reduction in mTORC1 signalling. This model is further supported by phytocannabinoids increasing IP_6_ production, which is independent of PI3K/PKB and mTORC1 activity (Maag et al., 2011). However, although our model suggests that phytocannabinoid activation of IPMK is PI3K/PKB independent, PI3K/PKB signalling remains necessary for the activation of mTORC1 that is consistent with earlier suggestions (Giacoppo et al., 2017), because pharmacological inhibition of PI3K activity and ablation of PKB activity (in *PKBA* ^*−*^ *:PKGB* ^*−*^ cells) lead to a block of mTORC1 activation. This model provides novel mechanistic insight with both CBG and CBD regulating mTORC1 via enhancing IPMK activity in the presence of a functionally active PI3K/PKB signalling pathway.

Understanding potential impacts of CBG in a clinical setting is unclear due to limited availability of information on CBG content in treatments. In clinical trials of phytocannabinoid‐based medicines containing THC and CBD (Novotna et al., 2011), the levels of other cannabinoids such as CBG provided to patients remain unspecified. In contrast, a similar product used in a mouse model of Huntington's disease contained between 0.9% and 1.1% CBG (Valdeolivas, Sagredo, Delgado, Pozo, & Fernandez‐Ruiz, 2017). Crude phytocannabinoid extracts, also likely to contain CBG, show efficacy in MS treatment (Rog, Nurmikko, Friede, & Young, 2005) and a clinical trial of a whole plant cannabis‐based medicine containing THC and CBD (but without defined levels of CBG) demonstrated successful treatment of muscle spasticity in pwMS (Collin et al., 2007). It is also worth noting that different cannabis strains demonstrate varying levels of CBG (De Backer et al., 2009). Thus, results presented here suggest that CBG should be further investigated for mTORC1 pathway regulation in this context.

This study provides insight into the mechanisms of action of phytocannabinoids, potentially relevant in the treatment of MS and underlying signalling. Autoimmunity drives MS pathogenesis, promoting immune cell‐induced neuroinflammation, myelin degradation and reactive changes in glia and axonal destruction (Bar‐Or et al., 2003). In addition, much evidence indicates that the innate immune system has a defined role in the progression and/or aetiology of MS (O'Brien et al., 2008) and that the PI3K/PKB/mTORC1 pathway provides a potentially relevant target pathway in MS (Mammana et al., 2018). Assuming that employing separate blots for total and phosphor‐4EBP1 provides an accurate readout of mTORC1 activity, we find that specific phytocannabinoids reduce mTORC1 signalling in immune cells (PBMCs), which is elevated during the proinflammatory activation of these cells (Gao et al., 2015) and therefore, our findings provide insight into a potential novel cellular target for phytocannabinoids in immune cells that may relate to MS treatment. In addition, we find that this effect is not seen in healthy individuals and is therefore evidence of a disease‐specific effect of immune cell activation.

Despite a range of proposed mechanisms for the therapeutic effects of phytocannabinoids, this study supports a mechanism via mTORC1 function. However, adenosine plays a key role in regulating AMP‐activated protein kinase (AMPK) activity and thus mTORC1 regulation (Ling et al., 2020), and extracellular adenosine treatment of a human‐derived cancer cell line reduced mTORC1‐dependent p4EBP1 levels (Choi et al., 2019). Similarly, the CBD‐dependent reduction in glycine and the one‐carbon cycle function (Perry et al., 2019) also impacts on adenosine levels and hence mTORC1 signalling (Boison, 2016). Finally, TRPV1 signalling has also been implicated in downstream regulation of mTORC1 activity (Maiese, 2017). Thus, further research into these existing targets and mechanisms will be necessary to establish if they also provide an impact on mTORC1 regulation.

This study proposes a mechanism of CBD and CBG through IPMK to regulate mTORC1 activity dependent upon activation state of the signalling pathway. Future studies may seek to validate this mechanism of both CBD and CBG in models of MS and psychosis and for the treatment of other mTOR‐dependent conditions. Our data also suggest that knowledge of CBG levels in cannabinoid extracts may be important to monitor various therapeutic roles.

## AUTHOR CONTRIBUTION

J.L.D.‐O., J.S., C.J.P. and E.C.W. carried out the *Dictyostelium* work. J.L.D.‐O. carried out the mouse embryonic fibroblast work. Y.D. and A.S. carried out the higher order phosphoinositide analysis. J.K.F., L.C. and E.D. carried out the PBMC work. R.S.B.W. conceived the project, supervised the work and, with J.L.D.‐O, wrote the paper. All authors contributed to and have approved the final manuscript.

## DECLARATION OF TRANSPARENCY AND SCIENTIFIC RIGOUR

This declaration acknowledges that this paper adheres to the principles for transparent reporting and scientific rigour of preclinical research as stated in the *BJP* guidelines for Natural Products Research, Design and Analysis and Immunoblotting and Immunochemistry, and as recommended by funding agencies, publishers and other organisations engaged with supporting research.

## Supporting information


**Figure S1.** Exemplar western blot analysis of *D._discoideum* and Human PBMC extracts showing p4EBP1 and total 4EBP1 antibody specificity. A. Full unedited gel relating to Figure 3E. Primary antibody against p4EBP1(p4EBP1(Thr37/46) rabbit, Cell SignallingTechnology, 9,459). Loading control (MCCC1) streptavidin (Streptavidin, AlexaFluor™ 680 conjugate, ThermoFisher, S21378). B. and C. Full unedited gels relating to Figure 7. Primary antibody against p4EBP1 (p4EBP1(Thr37/46) rabbit, cell signalling technology, 9,459). Primary antibody against total 4EBP1 (NEB,4923). Loading control actin (Sigma, A1978).
**Figure S2**. CBG does not affect development of wild type *D. discoideum.* Wild type *D. discoideum* cells, developed for 24 hours under starvation conditions form a field of mature fruiting bodies viewed from above, scale bar 1 mm for top view. In the presence of CBG (20 μM), development of the fruiting was unaffected. (Side view fruiting bodies in Figure 2).
**Figure S3**. *Homo sapiens* and *D.discoideum* IPMK protein alignment. Proteins were aligned using Clustal W. Amino acid conservation is visualised using: ‘*’indicating a position with a conserved amino acid residue, ‘:’indicating a position with conservation between two amino acid with strongly similar properties,.’indicating a position with conservation between two amino acid with weakly similar properties.
**Figure S4**. IPMK levels in the mutant and overexpression of IPMK. A: qPCR analysis of REMI mutant resistant to CBG to determine how *ipmk* mRNA levels differed in the mutant compared to wild type, n = 3. Data were not normally distributed (according to Shapiro–Wilks), therefore a Mann Whitney test was carried out to test for significance, * ‐ p ≤ 0.05. B: *D. discoideum* (*IPMK+*) and *H. sapiens IPMK+*‐RFP (*hIPMK*+) plasmids were created. This led to the increased expression of both proteins. Western blot analysis was used to determine that these proteins were overexpressed. Loading control protein is MCCC1. The housekeeping gene used was Ig7 (DDB_G0294034) and the method used to calculate fold‐change was ΔΔ‐Ct.
**Figure S5**. Analysis of the effect of CBG on growth in the *IPMK+* and *hIPMK+* cell lines. *D. discoideum* mutant cell lines were grown in the presence of increasing concentrations of CBG in shaking culture for one week. Growth is calculated as a % of each cell line in solvent only conditions. A: *IPMK+* (n = 6), used to calculate secondary plot in Figure 3. B: *hIPMK+* (n = 7), used to calculate secondary plot in Figure 3.
**Figure S6**. Docking analysis of *D.discoideum* and human IPMK with CBG and CBD. For molecular docking analyses predictions of tertiary protein structures were generated and Swiss Dock was used to detect potential binding sites of CBG and CBD. A. The most likely binding site showing the lowest ΔG (Gibbs free energy). For the *Dictyostelium* IPMK, but not for the human IPMK, similar binding sites were predicted for CBG and CBD. B. The potential direct binding sites for both phytocannabinoids on both proteins. C. Alignment of potential binding sites on both protein.
**Figure S7**. Analysis of the effect of CBG on growth in the *PKBA‐* and *PKBA‐IPMK+* cell lines. *D. discoideum* mutant cell lines were grown in the presence of increasing concentrations of CBG in shaking culture for one week. Growth shown as a % of each cell line in solvent only conditions. A: *PKBA‐* (n = 7), used to calculate secondary plot in Figure 4. B: *PKBA‐IPMK+* (n = 5), used to calculate secondary plot in Figure 4.
**Figure S8**. Phospho‐GEFS western blot analysis suggests cannabinoids do not alter mTORC2 activity in *D. discoideum*. Wild type (WT) *D. discoideum* cells were treated with CBG or CBD (0.25 μM for 1 hour) or solvent only control (DMSO, −) prior to Western analysis. The *PKBA*‐*PKGB*‐ mutant was used as a negative control and pGEFS levels were determined using an antibody against p‐PKB substrate (110 kDa band; Kamimura et al., 2008) with MCCC1 as a loading control. Data were not normally distributed as tested by Shapiro–Wilk's and significance was assessed by Kruskal‐Wallis test with a with Dunn's Multiple Comparison Post‐test, ** p < 0.01, n = 7. Graph shows mean ± SEM
**Figure S9**. Phospho‐ and total‐4EBP1 western blot analysis showing cannabinoids upregulate mTORC1 activation in the presence of IPMK through PI3K‐dependent signalling in MEFs. These graphs relate to Figure 6. Mouse embryonic fibroblast (MEF) cells were exposed to 4 μM CBG or CBD or vehicle (−) control for 24 hours. Cells were then collected and analysed by western blot for total and phosphorylated 4EBP1 levels, with actin as the control protein. Data were normally distributed with Shapiro‐Wilks and significance was tested using a One ‐Way ANOVA with Dunnett's Multiple Comparison Test. A, C, E: Total 4EBP1 content of MEF cells treated with 4 μM CBG or CBD was measured using western blot analysis. B, D, F: Phosphorylation of 4EBP1 in MEF cells treated with 4 μM CBG or CBD was measured using western blot analysis. A: CBG or CBD of WT MEF cells had no effect on total 4EBP1 levels compared to vehicle only treatment (−), n = 9; DF:2,25, F = 7.0. B: CBG or CBD of WT MEF cells significantly increased phosphorylation of 4EBP1 compared to untreated, n = 9;DF:2,26, F = 5.7. C: CBG or CBD of MEF cells lacking IPMK (*IPMK−/−*) had no effect on total 4EBP1 levels compared to vehicle only treatment (−), n = 9; DF:2,24, F = 0.7. D: CBG or CBD of MEF cells lacking IPMK (*IPMK−/−*) had no effect on phosphorylation of 4EBP1 levels compared to vehicle only treatment (−), n = 9; DF:2,26, F = 0.1. E: CBG or CBD of MEF cells lacking PI3K activity (treated with PI3K inhibitor: 10 μM Pictilisib) had no effect on total 4EBP1 levels compared to vehicle only treatment (−), n = 9; DF:2,24, F = 3.2. F: CBG or CBD of MEF cells lacking PI3K activity (treated with PI3K inhibitor: 10 μM Pictilisib) had no effect on phosphorylation of 4EBP1 levels compared to vehicle only treatment (−), n = 9; DF:2,24, F = 0.7.
**Figure S10**. Rapamycin treatment of peripheral blood mononuclear cells (PBMCs): A and B: To determine if mTORC1 activity in primary PBMCs could be examined, cells were treated with the mTOR inhibitor, rapamycin (200 nM). In cells from both healthy individuals and individuals with multiple sclerosis rapamycin significantly decreased the proportion of phosphorylated 4EBP1 compared to total 4EBP1. C and D: To determine if mTORC1 activity in primary PBMCs could be examined, cells were treated with rapamycin (200 nM). In cells from both healthy individuals and individuals with multiple sclerosis, rapamycin did not significant alter p4EBP1 levels. D and E: Rapamycin (200 nM) treatment of cells from both healthy individuals and individuals with multiple sclerosis rapamycin significantly increased total 4EBP1.

## Data Availability

The data that support the findings of this study are available from the corresponding author upon reasonable request. Some data may not be made available because of privacy or ethical restrictions.
