# Supplementary material for: Phytocannabinoid‐dependent mTORC1 regulation is dependent upon inositol polyphosphate multikinase activity
Source: Br J Pharmacol. 2021 Jan 18;178(5):1149–63. doi: 10.1111/bph.15351 (PMC9328663; doi:10.1111/bph.15351)
Supplement: Supplementary file 1 — Figure S1. Exemplar western blot analysis of D._discoideum and Human PBMC extracts showing p4EBP1 and total 4EBP1 antibody specificity. A. Full unedited gel relating to Figure 3E. Primary antibody against p4EBP1(p4EBP1(Thr37/46) rabbit, Cell SignallingTechnology, 9,459). Loading control (MCCC1) streptavidin (Streptavidin, AlexaFluor™ 680 conjugate, ThermoFisher, S21378). B. and C. Full unedited gels relating to Figure 7. Primary antibody against p4EBP1 (p4EBP1(Thr37/46) rabbit, cell signalling technology, 9,459). Primary antibody against total 4EBP1 (NEB,4923). Loading control actin (Sigma, A1978). Figure S2. CBG does not affect development of wild type D. discoideum. Wild type D. discoideum cells, developed for 24 hours under starvation conditions form a field of mature fruiting bodies viewed from above, scale bar 1 mm for top view. In the presence of CBG (20 μM), development of the fruiting was unaffected. (Side view fruiting bodies in Figure 2). Figure S3. Homo sapiens and D.discoideum IPMK protein alignment. Proteins were aligned using Clustal W. Amino acid conservation is visualised using: ‘*’indicating a position with a conserved amino acid residue, ‘:’indicating a position with conservation between two amino acid with strongly similar properties,.’indicating a position with conservation between two amino acid with weakly similar properties. Figure S4. IPMK levels in the mutant and overexpression of IPMK. A: qPCR analysis of REMI mutant resistant to CBG to determine how ipmk mRNA levels differed in the mutant compared to wild type, n = 3. Data were not normally distributed (according to Shapiro–Wilks), therefore a Mann Whitney test was carried out to test for significance, * ‐ p ≤ 0.05. B: D. discoideum (IPMK+) and H. sapiens IPMK+‐RFP (hIPMK+) plasmids were created. This led to the increased expression of both proteins. Western blot analysis was used to determine that these proteins were overexpressed. Loading control protein is MCCC1. The housekeeping gene u [file BPH-178-1149-s001.pdf]

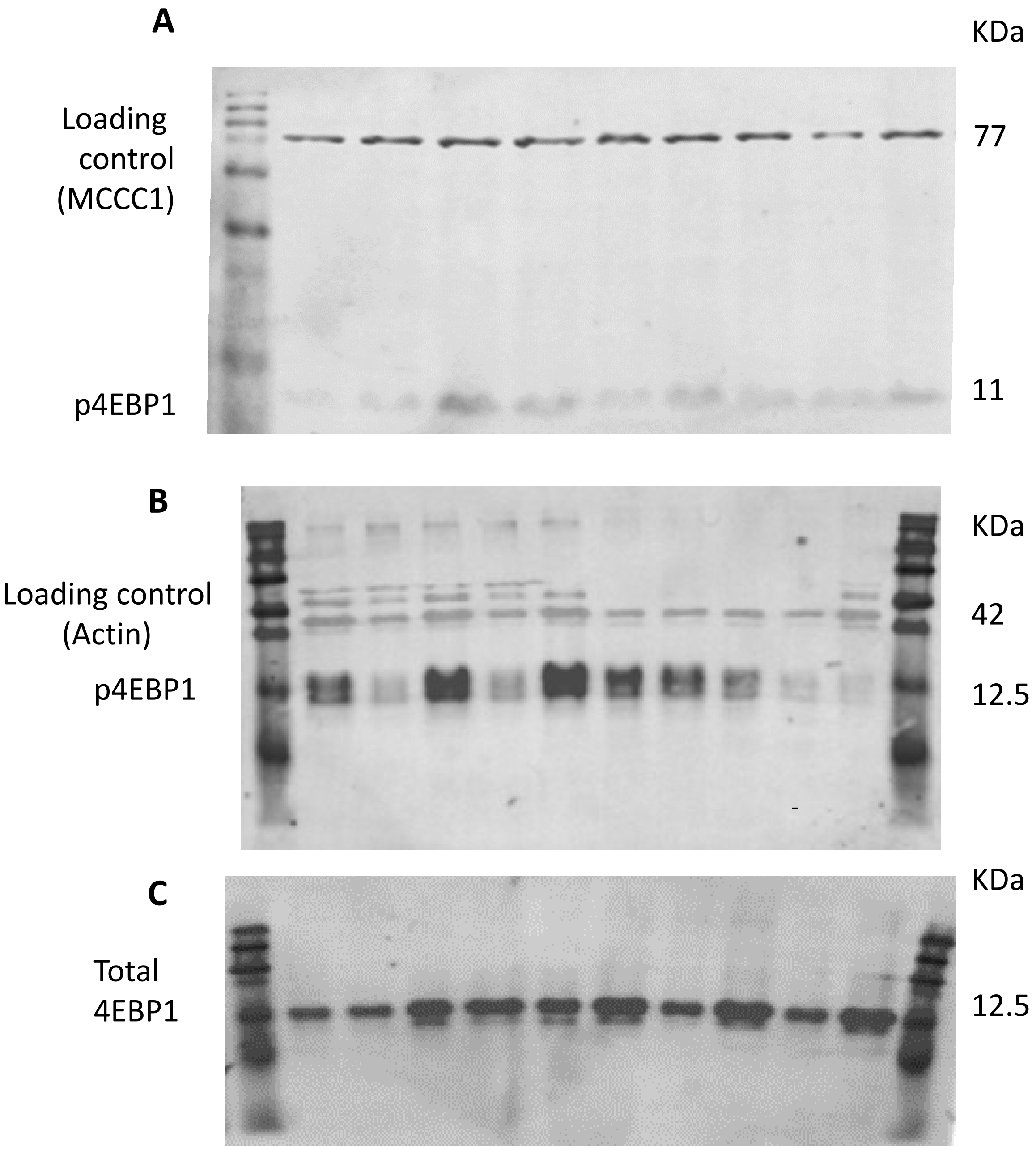

Supplementary Fig 1: Exemplar Western blot analysis of *D. discoideum* and Human PBMC extracts showing p4EBP1 and total 4EBP1 antibody specificity. A. Full unedited gel relating to Figure 3E. Primary antibody against p4EBP1 (p4EBP1 (Thr37/46) rabbit, Cell Signalling Technology, 9459). Loading control (MCCC1) streptavidin (Streptavidin, Alexa Fluor™ 680 conjugate, Thermo Fisher, S21378). **B. and C**. Full unedited gels relating to Figure 7. Primary antibody against p4EBP1 (p4EBP1(Thr37/46) rabbit, cell signalling technology, 9459). Primary antibody against total 4EBP1 (NEB, 4923). Loading control actin (Sigma, A1978).

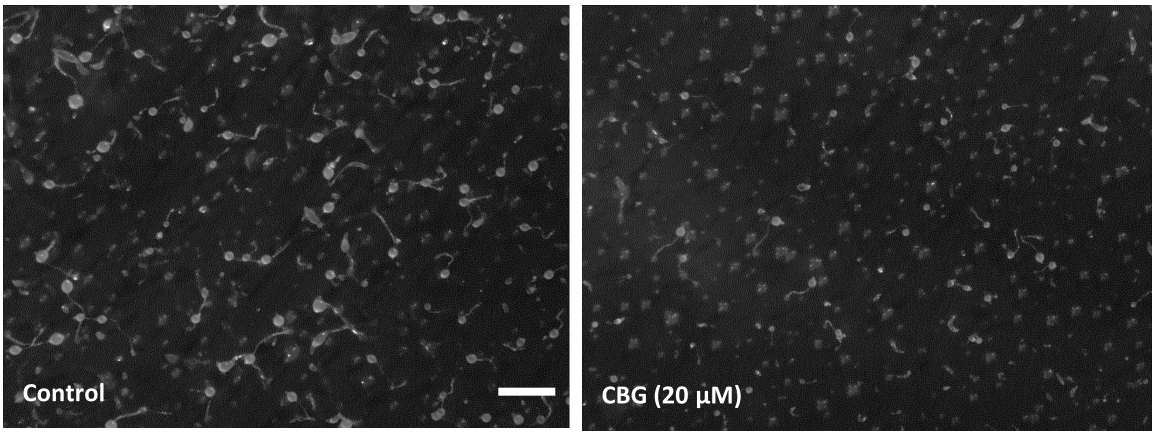

Supplementary Figure 2: CBG does not affect development of wild type *D. discoideum*. Wild type *D. discoideum* cells, developed for 24 hours under starvation conditions form a field of mature fruiting bodies viewed from above, scale bar 1 mm for top view. In the presence of CBG (20 µM), development of the fruiting was unaffected. (Side view fruiting bodies in Fig 2).

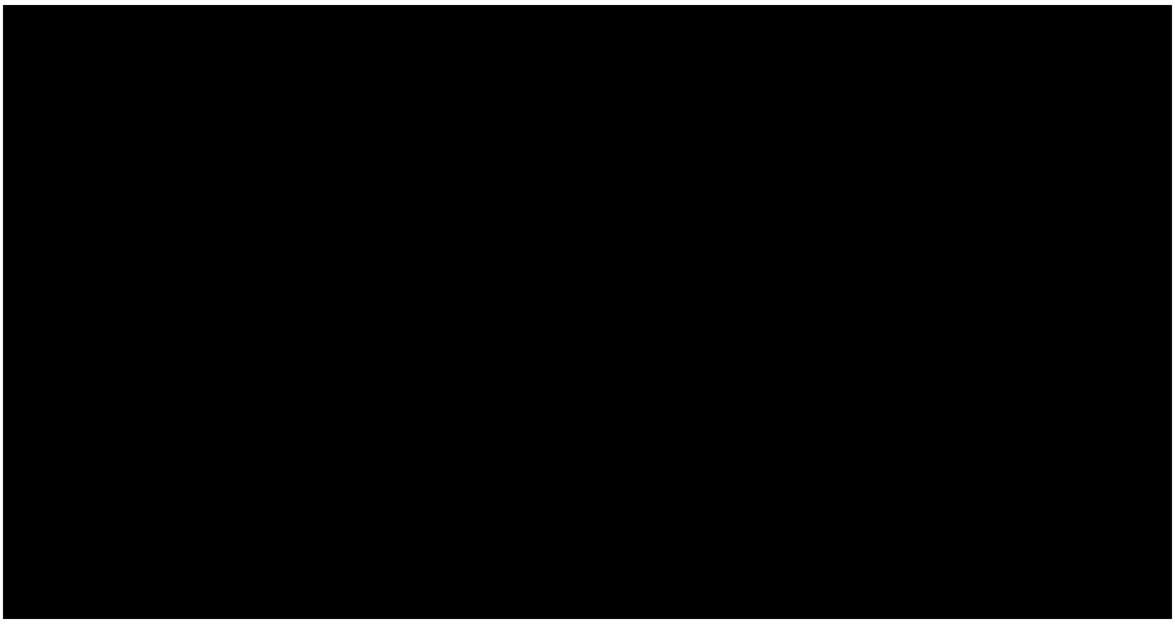

Supplementary Figure 3: *Homo sapiens* and *D. discoideum* IPMK protein alignment. Proteins were aligned using Clustal W. Amino acid conservation is visualised using: ‘*’ indicating a position with a conserved amino acid residue, ‘:’ indicating a position with conservation between two amino acid with strongly similar properties, .’ indicating a position with conservation between two amino acid with weakly similar properties.

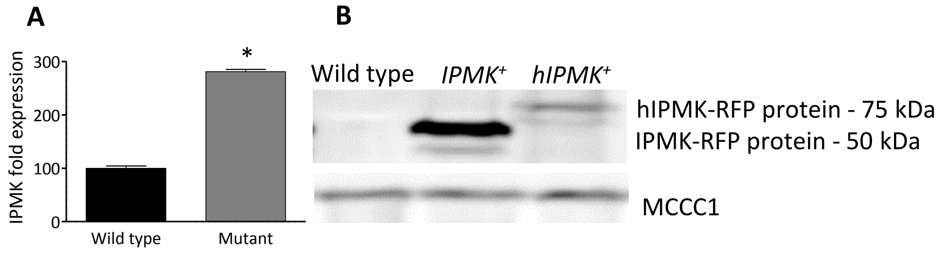

Supplementary Figure 4: IPMK levels in the mutant and overexpression of IPMK. A: qPCR analysis of REMI mutant resistant to CBG to determine how *ipmk* mRNA levels differed in the mutant compared to wild type, n=3. Data were not normally distributed (according to Shapiro-Wilks), therefore a Mann Whitney test was carried out to test for significance, * - p≤0.05. **B:** *D. discoideum* (*IPMK*$^{+}$) and *H. sapiens IPMK*$^{+}$-RFP (*hIPMK*$^{+}$) plasmids were created. This led to the increased expression of both proteins. Western blot analysis was used to determine that these proteins were overexpressed. Loading control protein is MCCC1. The housekeeping gene used was Ig7 (DDB_G0294034) and the method used to calculate fold-change was ΔΔ-Ct.

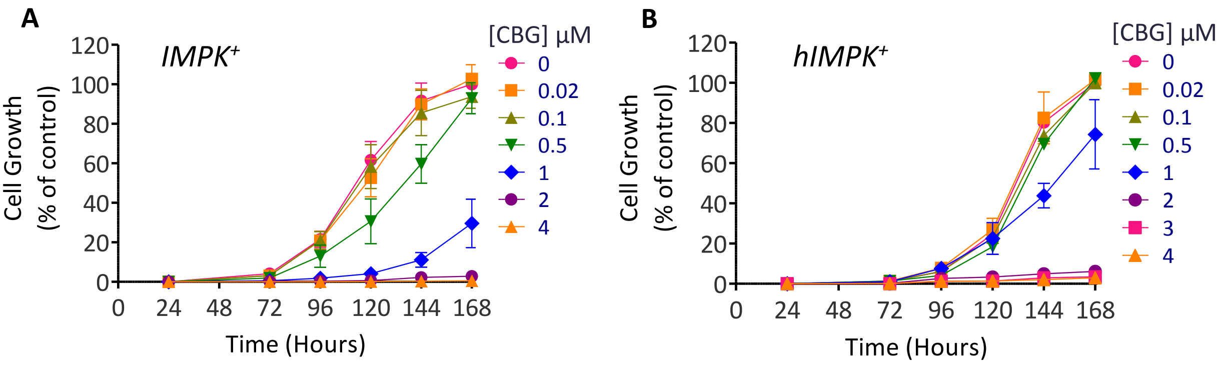

Supplementary Figure 5: Analysis of the effect of CBG on growth in the *IPMK*+ and *hIPMK*+ cell lines. *D. discoideum* mutant cell lines were grown in the presence of increasing concentrations of CBG in shaking culture for one week. Growth is calculated as a % of each cell line in solvent only conditions. **A:** *IPMK*+ (n=6), used to calculate secondary plot in Figure 3. **B:** *hIPMK*+ (n=7), used to calculate secondary plot in Figure 3.

A

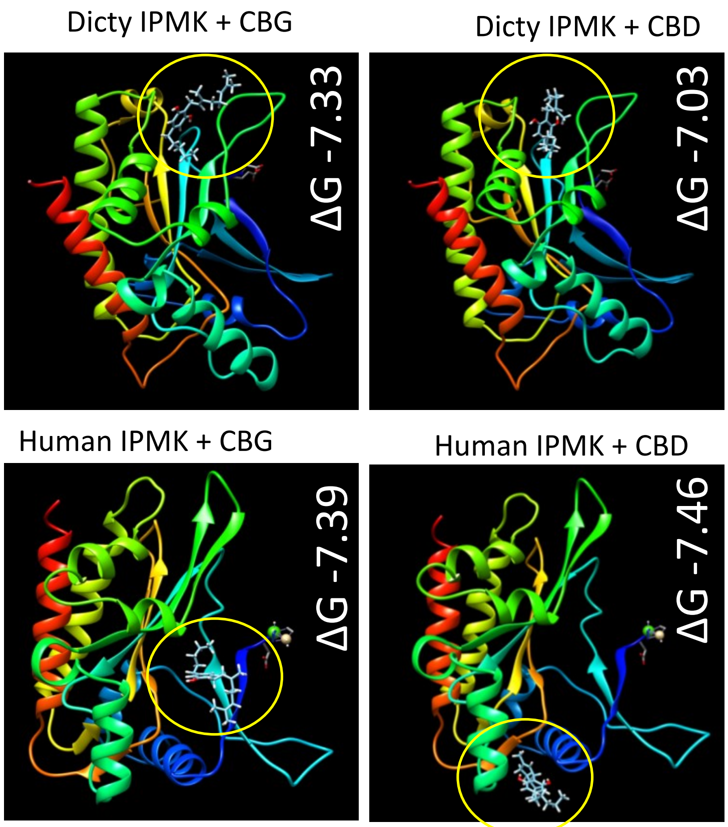

B

	potential direct binding
Dicty IPMK + CBG	Glu 148, Thr150, Trp175, Arg172, Thr73
Human IPMK + CBG	Leu179, Glu194, Val389
Dicty IPMK + CBD	Glu148, Arg172
Human IPMK + CBD	Glu88

C

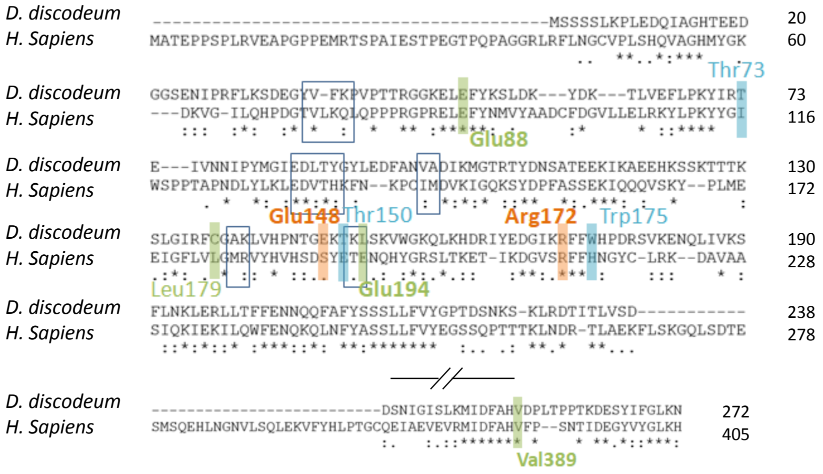

Supplementary Figure 6: Docking analysis of *D. discoideum* and human IPMK with CBG and CBD. For molecular docking analyses predictions of tertiary protein structures were generated and SwissDock was used to detect potential binding sites of CBG and CBD. **A.** The most likely binding site showing the lowest ΔG (Gibbs free energy). For the *Dictyostelium* IPMK, but not for the human IPMK, similar binding sites were predicted for CBG and CBD. **B.** The potential direct binding sites for both phytocannabinoids on both proteins. **C.** Alignment of potential binding sites on both protein.

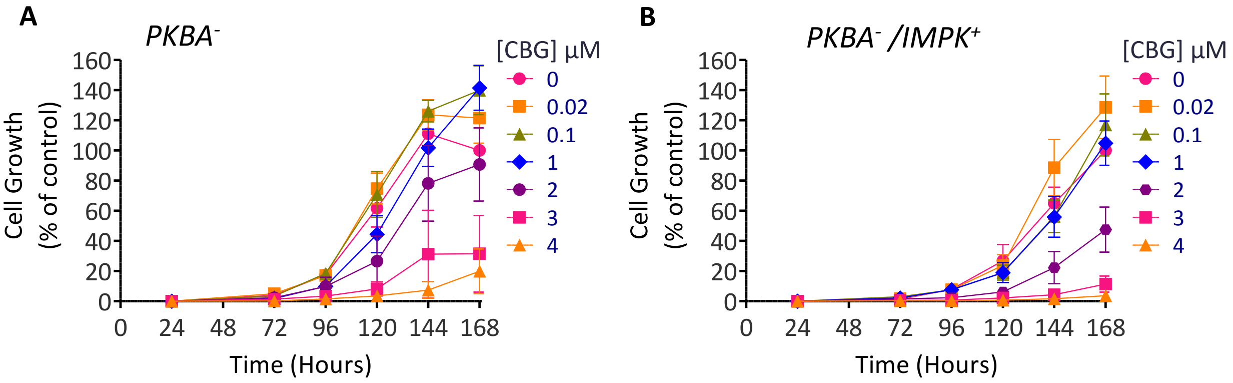

Supplementary Figure 7: Analysis of the effect of CBG on growth in the *PKBA⁻* and *PKBA⁻IPMK⁺* cell lines. *D. discoideum* mutant cell lines were grown in the presence of increasing concentrations of CBG in shaking culture for one week. Growth shown as a % of each cell line in solvent only conditions. **A:** *PKBA⁻* (n=7), used to calculate secondary plot in Figure 4. **B:** *PKBA⁻IPMK⁺* (n=5), used to calculate secondary plot in Figure 4.

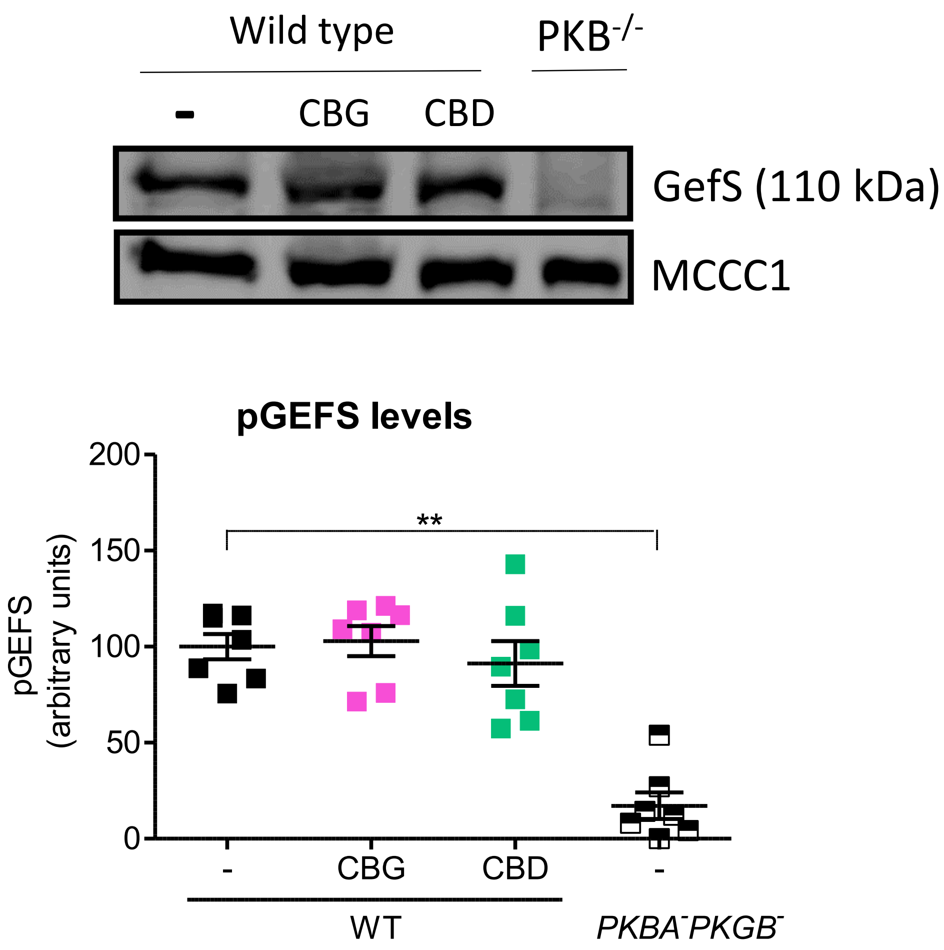

Supplementary Figure 8: Phospho-GEFS western blot analysis suggests cannabinoids do not alter mTORC2 activity in *D. discoideum*. Wild type (WT) *D. discoideum* cells were treated with CBG or CBD (0.25 µM for 1 hour) or solvent only control (DMSO, -) prior to Western analysis. The *PKBA⁻PKGB⁻* mutant was used as a negative control and pGEFS levels were determined using an antibody against p-PKB substrate (110 kDa band; Kamimura et al., 2008) with MCCC1 as a loading control. Data were not normally distributed as tested by Shapiro-Wilk's and significance was assessed by Kruskal-Wallis test with a with Dunn's Multiple Comparison Post-test, ** $p<0.01$, n = 7. Graph shows mean ± SEM

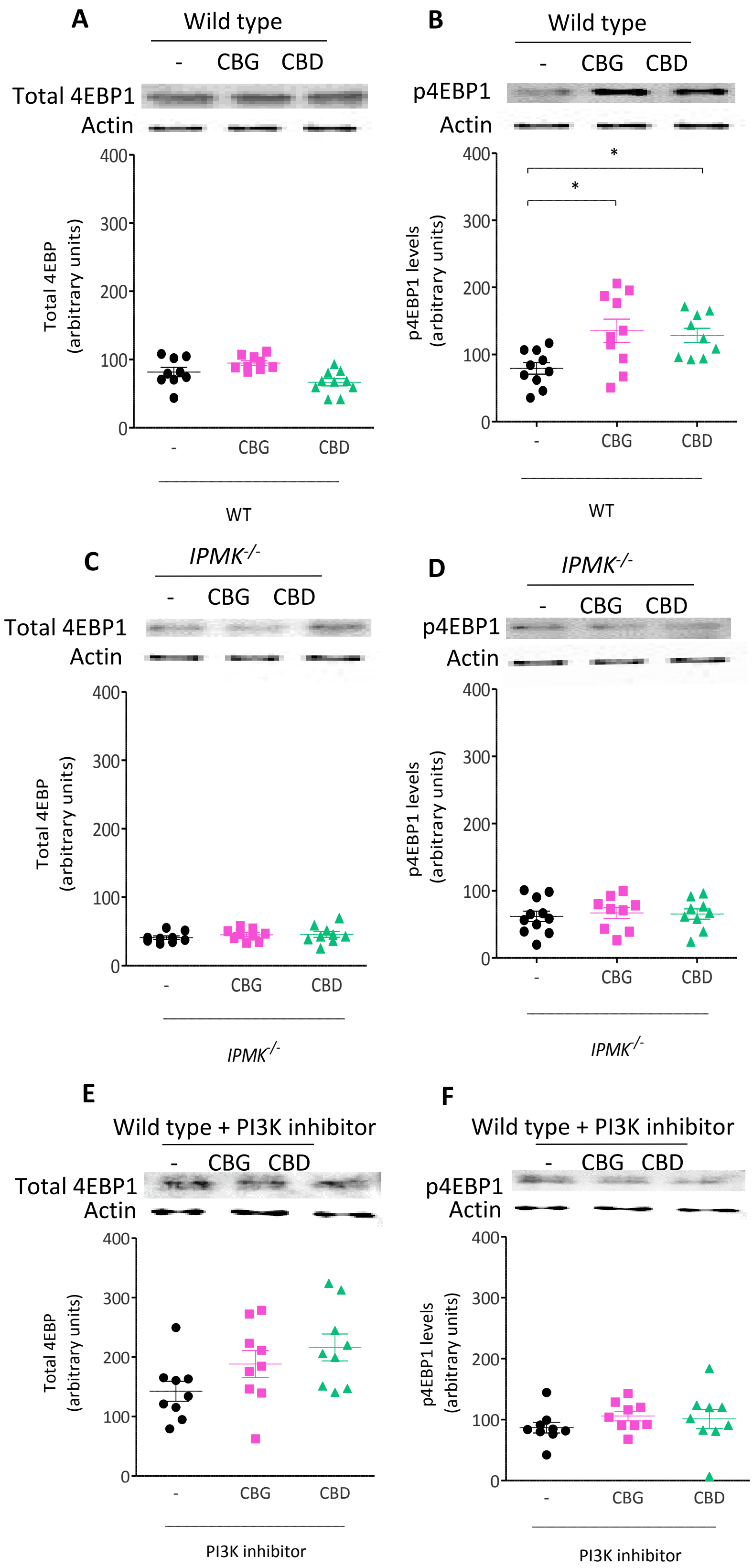

Supplementary Figure 9: Phospho- and total-4EBP1 western blot analysis showing cannabinoids upregulate mTORC1 activation in the presence of IPMK through PI3K-dependent signalling in MEFs. These graphs relate to Figure 6. Mouse embryonic fibroblast (MEF) cells were exposed to 4 µM CBG or CBD or vehicle (-) control for 24 hours. Cells were then collected and analysed by western blot for total and phosphorylated 4EBP1 levels, with actin as the control protein. Data were normally distributed with Shapiro-Wilks and significance was tested using a One -Way ANOVA with Dunnett's Multiple Comparison Test. **A, C, E:** Total 4EBP1 content of MEF cells treated with 4 µM CBG or CBD was measured using western blot analysis. **B, D, F:** Phosphorylation of 4EBP1 in MEF cells treated with 4 µM CBG or CBD was measured using western blot analysis. **A:** CBG or CBD of WT MEF cells had no effect on total 4EBP1 levels compared to vehicle only treatment (-), n=9; DF:2,25, F=7.0. **B:** CBG or CBD of WT MEF cells significantly increased phosphorylation of 4EBP1 compared to untreated, n=9;DF:2,26, F=5.7. **C:** CBG or CBD of MEF cells lacking IPMK (*IPMK*$^{-/-}$) had no effect on total 4EBP1 levels compared to vehicle only treatment (-), n=9; DF:2,24, F=0.7. **D:** CBG or CBD of MEF cells lacking IPMK (*IPMK*$^{-/-}$) had no effect on phosphorylation of 4EBP1 levels compared to vehicle only treatment (-), n=9; DF:2,26, F=0.1. **E:** CBG or CBD of MEF cells lacking PI3K activity (treated with PI3K inhibitor: 10 µM Pictilisib) had no effect on total 4EBP1 levels compared to vehicle only treatment (-), n=9; DF:2,24, F=3.2. **F:** CBG or CBD of MEF cells lacking PI3K activity (treated with PI3K inhibitor: 10 µM Pictilisib) had no effect on phosphorylation of 4EBP1 levels compared to vehicle only treatment (-), n=9; DF:2,24, F=0.7.

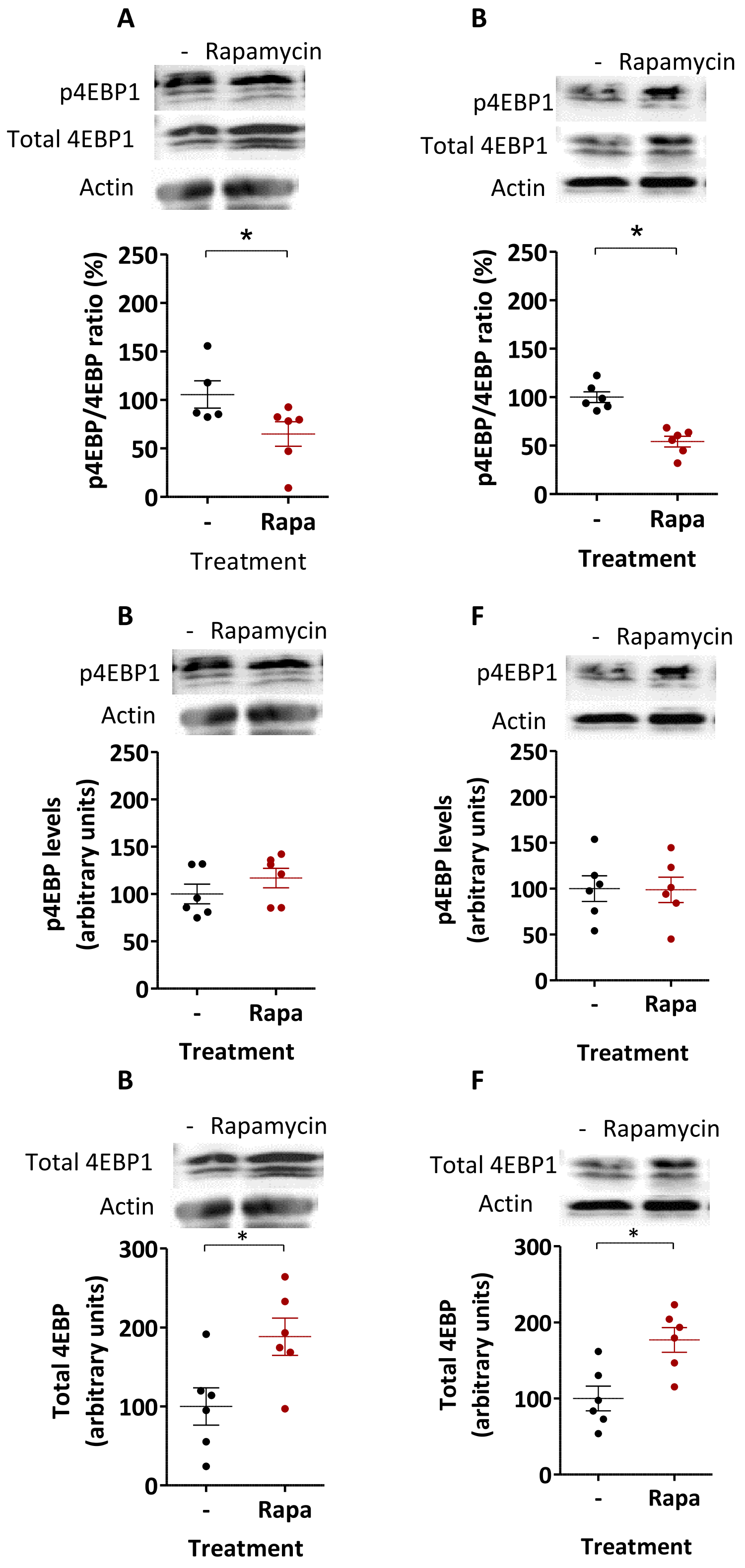

Supplementary Figure 10: Rapamycin treatment of PBMCs: A and B: To determine if mTORC1 activity in primary PBMCs could be examined, cells were treated with the mTOR inhibitor, rapamycin (200 nM). In cells from both healthy individuals and individuals with multiple sclerosis rapamycin significantly decreased the proportion of phosphorylated 4EBP1 compared to total 4EBP1. **C and D:** To determine if mTORC1 activity in primary PBMCs could be examined, cells were treated with rapamycin (200 nM). In cells from both healthy individuals and individuals with multiple sclerosis, rapamycin did not significant alter p4EBP1 levels. **D and E:** Rapamycin (200 nM) treatment of cells from both healthy individuals and individuals with multiple sclerosis rapamycin significantly increased total 4EBP1.
